# A heuristic information cluster search approach for precise functional brain mapping

**DOI:** 10.1002/hbm.24944

**Published:** 2020-02-07

**Authors:** Nima Asadi, Yin Wang, Ingrid Olson, Zoran Obradovic

**Affiliations:** ^1^ Department of Computer and Information Sciences, College of Science and Technology Temple University Philadelphia Pennsylvania; ^2^ Department of Psychology College of Liberal Arts, Temple University Philadelphia Pennsylvania; ^3^ Decision Neuroscience College of Liberal Arts, Temple University Philadelphia Pennsylvania

**Keywords:** algorithm design, data mining, functional magnetic resonance imaging, neuroimaging

## Abstract

Detection of the relevant brain regions for characterizing the distinction between cognitive conditions is one of the most sought after objectives in neuroimaging research. A popular approach for achieving this goal is the multivariate pattern analysis which is currently conducted through a number of approaches such as the popular searchlight procedure. This is due to several advantages such as being automatic and flexible with regards to size of the search region. However, these approaches suffer from a number of limitations which can lead to misidentification of truly informative regions which in turn results in imprecise information maps. These limitations mainly stem from several factors such as the fact that the information value of the search spheres are assigned to the voxel at the center of them (in case of searchlight), the requirement for manual tuning of parameters such as searchlight radius and shape, and high complexity and low interpretability in commonly used machine learning‐based approaches. Other drawbacks include overlooking the structure and interactions within the regions, and the disadvantages of using certain regularization techniques in analysis of datasets with characteristics of common functional magnetic resonance imaging data. In this article, we propose a fully data‐driven maximum relevance minimum redundancy search algorithm for detecting precise information value of the clusters within brain regions while alleviating the above‐mentioned limitations. Moreover, in order to make the proposed method faster, we propose an efficient algorithmic implementation. We evaluate and compare the proposed algorithm with the searchlight procedure as well as least absolute shrinkage and selection operator regularization‐based mapping approach using both real and synthetic datasets. The analysis results of the proposed approach demonstrate higher information detection precision and map specificity compared to the benchmark approaches.

## 1 INTRODUCTION

In the common form of functional magnetic resonance imaging (fMRI), the blood‐oxygen level dependent (BOLD) contrast is extracted as the response signal in order to measure neural activity in the brain (Huettel, Song, McCarthy, et al., 2004). Measurement of this response signal over time forms a time course corresponding to each voxel whose dimensions depend on the spatial resolution of the imaging device. Analysis and comparison of these time courses can reveal valuable knowledge regarding different neurological conditions among populations. Popular approaches for analyzing fMRI data can be broken down into two main categories: voxel‐wise univariate analysis, and multivoxel pattern analysis, also known as multivariate pattern analysis (MVPA; Wong, Palmeri, Rogers, Gore, & Gauthier, 2009; Norman, Polyn, Detre, & Haxby, 2006). The univariate analysis searches for correlations between psychological or physical status and the activation of single voxels while MVPA aims to detect patterns among conditions observed among combinations of multiple voxels (Davis et al., 2014). Unlike univariate analyses, MVPA approaches are designed to allow researchers to test how dispersed patterns of BOLD activation across multiple voxels relate to experimental conditions (Davis et al., 2014; Swearingen, 2015). One approach in multivoxel scheme is to compare and analyze spatially averaged (smoothed) BOLD activation across the entire regions of interest. Advantages of this approach include an increase in the signal to noise ratio as well as the consistency of the analysis among subjects can be noted (Jimura & Poldrack, 2012). However, spatial smoothing leads to significant loss of information about the patterns of activation within the regions of interest. This information includes the activities and dynamics within subregions which can provide valuable insight into their relation with different mental states (Gardumi et al., 2016; Stelzer, Chen, & Turner, 2013). This issue becomes more complex when dealing with larger regions of interest. Therefore, in order to capture such information, it is necessary to consider the BOLD activity in smaller spherical subsets (Etzel, Zacks, & Braver, 2013).

The question of identifying relevant regions with regards to specific conditions has prompted numerous studies during the recent decades. One of the most commonly employed approaches for this application is the searchlight method proposed by Kriegeskorte et al., which given the dimensions of a sphere window, performs a search across a brain region to detect the information of sets of neighboring voxels (Kriegeskorte & Bandettini, 2007; Kriegeskorte, Goebel, & Bandettini, 2006). In this multivariate approach, spatial patterns of activity within the search window are compared between two groups using statistical discriminant analysis or supervised machine learning approaches (Chen et al., 2011; Uddin et al., 2011). The search sphere (searchlight) is centered on every voxel, that is, the derived separability value for each voxel is derived from the discrimination score of its surrounding searchlight, not the voxel individually. Advantages of searchlight analysis include its automatic procedure, its ability in performing whole‐brain search without the need to specify brain regions, and its high interpretability and intuition.

However, the searchlight procedure suffers from multiple drawbacks which can lead to erroneous detection of informative voxels/regions. Etzel et al. discussed several issues with the searchlight method in detail which we briefly point out here (Etzel et al., 2013). One limitation of the searchlight procedure is that it can declare a subregion with a few highly informative voxels as informative, making detection of informative voxel clusters ambiguous. This issue becomes more prevalent with selection of larger search radii (Etzel et al., 2013). Moreover, choosing an appropriate search radius is essential, which depends on the shape and size of the region being searched. However, finding the discriminative subregion by a search over several possible search radius values is difficult especially when being applied to whole‐brain analysis. Aside from this issue, the shape of the searchlight can limit the detection of the subregions with the highest discrimination power. This is due to the fact that the searchlight is commonly in the shape of a sphere or a cube, which forces subregions with irregular shapes to fall between multiple searchlight positions. This issue can partially be relieved through assigning the searchlight sphere as small as possible at the expense of overfitting (Etzel et al., 2013). Another shortcoming with this method is the fact that assignment of a single searchlight radius might provide optimal results for one subregion, but does not guarantee similar results for many other regions. Consequently, finding the optimal searchlight radius for a large search space comprised of subspaces with varying anatomical characteristics is a challenging task. Tackling some of the mentioned issues requires further analysis while some issues are inherently irresolvable through the scope of the searchlight procedure.

Several other approaches have been proposed based on machine learning techniques to create models for automated decoding of cognitive states during recent years. A number of these techniques proposed using different variations of the least absolute shrinkage and selection operator (LASSO) family to develop a continuous feature evaluation process (Gramfort, Thirion, & Varoquaux, 2013; Ng & Abugharbieh, 2011; Shimizu et al., 2015; Toiviainen, Alluri, Brattico, Wallentin, & Vuust, 2014). However, the use of LASSO regularization in fMRI studies introduces several limitations. One of such constraints is the fact that in case of number of features *p* being larger than the number of examples *m*, LASSO selects *m* features at maximum (Tibshirani & Saunders, 2005). This is a critical drawback due to the fact that in fMRI studies, especially on voxel‐level analysis, it is very common that the number of subjects is far smaller than the number of features (voxels, or even regions of interest). Another drawback of LASSO is the fact that since it forces less important coefficients to be zero, it does not provide the information value of the features that have not been selected. Consequently, instead of creating an information spectrum, it points to a small subset of features that it finds to be more informative, which makes it less useful for researchers in fMRI studies due to loss of knowledge regarding majority of the brain areas. Moreover, using a more recent variation of LASSO which considers group structure named group LASSO requires disjoint subsets of the voxels to be predetermined. This limitation creates an issue similar to the searchlight analysis radius selection since the choice of size and structure of the groups of voxels changes the results of the feature space shrinkage. Also, the interpretability of performing a regularization‐based approach on the entire feature space is low. Another method for detecting biomarkers is the manifold learning suggested by Wolz, Aljabar, Hajnal, and Rueckert (2010). Despite its power in nonlinear classification of MR images and the consideration of spectral theory in dimensionality reduction, several parameters need to be fine‐tuned for it to achieve preferable results. These parameters include the optimal neighborhood size, the number of dimensions learned by the manifold, and the heat kernel parameter which the Laplacian eigenmap feature selection is sensitive to. Also, time complexity of the spectral embedding phase of manifold learning grows substantially with the size of neighborhood, making it less efficient for full‐brain analysis (Belkin & Niyogi, 2003). In another recent work, Varol et al. suggested a linear multivariate discriminative statistical mapping using least squares support vector machine (LS‐SVM) to achieve higher sensitivity and specificity in detecting group differences while preserving computational efficiency of the analysis (Varol, Sotiras, & Davatzikos, 2018). This approach can be used for both classification and regression problems and can employ various local learners based on the scale of the regions to be mapped. However, this method also requires the radius of the neighborhood as well as the LS‐SVM parameters (including the SVM slack variable C and any other possible kernel variables) to be tuned to ensure the best performance.

Another category of approaches in decoding the cognitive state of the brain is based on the principles of deep neural networks. Although family of techniques have been mostly used in lesion segmentation and functional connectivity analysis, a number of studies have also employed them to create a mapping or visualization of the spatial information of the regions. In this approach, the task of classifying the neurological conditions is treated similar to image classification where a model such as a convolutional neural network (CNN) is trained based on high resolution activation patterns in the brain. Deep learning‐based approaches require a large number (usually to the order of hundreds of thousands) of examples for effective training and parameter tuning. In order to transfer a pretrained CNN on fMRI data, a three‐dimensional CNN is required to be trained on a large set of imaging data to extract the necessary features for fMRI analysis (Hossain, Umar, Alsulaiman, & Muhammad, 2019; Jang, Plis, Calhoun, & Lee, 2017; Kamnitsas et al., 2017; Liu et al., 2018; Pinaya et al., 2016; Sarraf & Tofighi, 2016; Wang et al., 2019). However, using deep CNNs requires a certain level of expertise to interpret the high level features and to fine‐tune the network for the specific task of fMRI classification, and often comes with significant computational complexity (Heinsfeld, Franco, Craddock, Buchweitz, & Meneguzzi, 2018; Liu et al., 2018). Furthermore, Bjornsdotter et al. proposed an MVPA approach based on Monte‐Carlo sampling where information is combined across overlapping neighborhoods (Bjo¨rnsdotter, Rylander, & Wessberg, 2011). Despite its advantage in increasing the stability, this approach does not precisely provide the significance of each voxel in characterizing a certain condition.

In conclusion, development of new analytical models which tackles the above‐mentioned issues while retaining the beneficial aspects of those approaches is essential for the critical task of automatically discovering the information of different regions regarding certain neurological conditions. In this study, we propose a new approach for extracting features with voxel‐level precision. The aim of this approach is to provide an interpretable mapping of information clusters where the spatial proximity and the interactions between the voxel‐level regions are taken into account without the requirement of parameter tuning. Through a completely data‐driven search, the proposed approach achieves this goal while increasing the classification accuracy at the same time. Through empirical results on a real fMRI dataset as well as synthetic data, we compare the performance of the proposed algorithm with the searchlight methods. We explain the experimental results as well as the suggested methodology in more detail in the next sections.

## 2 METHODOLOGY

The objective of the proposed methodology is to create the information map of the brain (or regions of interest) with regards to a certain neurological status, for example, a cognitive disorder, age, activation pattern differences during different tasks, and so forth. In other words, given two (or more) populations, the goal is to discover the level at which the information based on BOLD activation of brain regions differ between groups, which in this study we define as the discriminant score of the region (note that we use the terms discriminant score and information interchangeably in order to preserve consistency with the related literature). We also define a cluster as a group of neighboring voxels whose size can span from one voxel to the entire area to be searched, and a search space as the region of brain that we intend to explore (search) and investigate in order to detect “informative” subregions. Furthermore, since each voxels activity level is treated as a feature, the terms “voxel” and “feature” in this article, bear the same meaning. Generally, the input to the proposed approach is a data matrix where each row corresponds to one subject, and each column corresponds to a voxel in the search space. Therefore, each element in the data matrix contains the activation or BOLD value of a voxel in the search space averaged over time. The output of the proposed algorithm is a set of information clusters where each cluster is assigned an information score. To simplify reference to the proposed algorithm, we refer to it as ICS, which is the acronym for information cluster search.

### 2.1 The proposed algorithm

To extract the informative clusters within a region of interest we propose an algorithm which traverses the search space based on an information‐based heuristic, and outputs the discovered information clusters and their measured discriminant scores after termination. This process resembles greedy search algorithms which pick the “best” neighbor according to a heuristic, which in this case is the discriminant score of the cluster of voxels (Dechter & Pearl, 1985; DeVore & Temlyakov, 1996). However, unlike common greedy algorithm procedures, the proposed approach reviews the searched clusters and prunes the redundant voxels after each expansion step to increase its precision and reduce the risk of falling in local optima.

In general, ICS includes two steps, expansion step, and pruning step, which are periodically performed for each information cluster. During the expansion step, ICS performs a search starting from a voxel *V* to detect its immediate neighbors whose pairing with *V* (adding it as a feature to the feature set that includes *V*) enhance its power in distinguishing between the classes of data. This process is the maximum relevance approach in feature selection. Then, the pruning step starts in which a redundancy detection is performed on the newly created cluster to remove the redundant voxels and further optimize the selected cluster. This analysis is performed due to the fact that addition of new features to a feature group can introduce new redundancies, that is, the Markov blanket (MB) of the target variable defined as the optimal set of attributes to predict it can change due to the influence of the newly added feature (Aliferis, Statnikov, Tsamardinos, Mani, & Koutsoukos, 2010; Koller & Sahami, 1996). After the redundancy procedure, similar expansion process is performed on the pruned cluster, meaning that the neighboring voxels of the entire remaining cluster after redundancy analysis are examined to find the useful voxels to add to the cluster. The expansion and pruning process are repeated until there are no neighboring voxels left whose addition to the detected cluster is helpful. Note that ICS avoids removing newly added voxels or admitting voxels that have been found redundant in the last step in order to avoid falling in an infinite loop. The detailed pseudocode of the algorithm is provided in the Supplementary Information. Searching the neighborhood of the entire cluster alleviates the issue of falling in local optima in greedy algorithms (Preparata & Shamos, 2012; Tan, He, & Aaron, 2006). On the other hand, the proposed procedure relaxes the requirement of performing a search through all possible combinations while increasing the spatial precision of search by investigating voxel‐level resolutions. In general, the steps of the proposed algorithm go as followed (details are provided in the Supplementary Information):

*Step 1*: Start from voxel *v*_*s*_ and measure the relevance score of its conjugation with each of its immediate neighbors *v*_*n*_ ∈ *V*_neighbors_ one by one. Select the subset of neighbors *V*_pos_ ⊂ *V*_neighbor_ whose addition to *v*_*s*_ provides larger information value than the individual information quality of *v*_*s*_ (Equation (1)). Then, combine *v*_*s*_ and *V*_pos_ to create the information cluster IC = *v*_*s*_ ∪ *V*_pos_.

*Step 2*: Search each voxel adjacent to IC, and admit the neighboring voxels whose addition to *IC* enhances its relevance score, (similar to step one, but for the entire *IC*).

*Step 3*: Perform the feature redundancy analysis on *IC* and remove its redundant voxels (except the newly added voxels).

*Step 4*: Repeat Steps 2 (except the newly removed voxels) and 3 to expand and prune the cluster until there is no new neighbor whose addition to *IC* increases its score. Save *IC* and its information in the output variable.

*Step 5*: Start from the voxel next to *v*_*s*_ and follow Steps 1–4.

*Step 6*: When Steps 1–4 are performed for every voxel as the starting voxel in the search space, terminate the algorithm and output the set of discovered information clusters and their information value.

**Figure 1:**
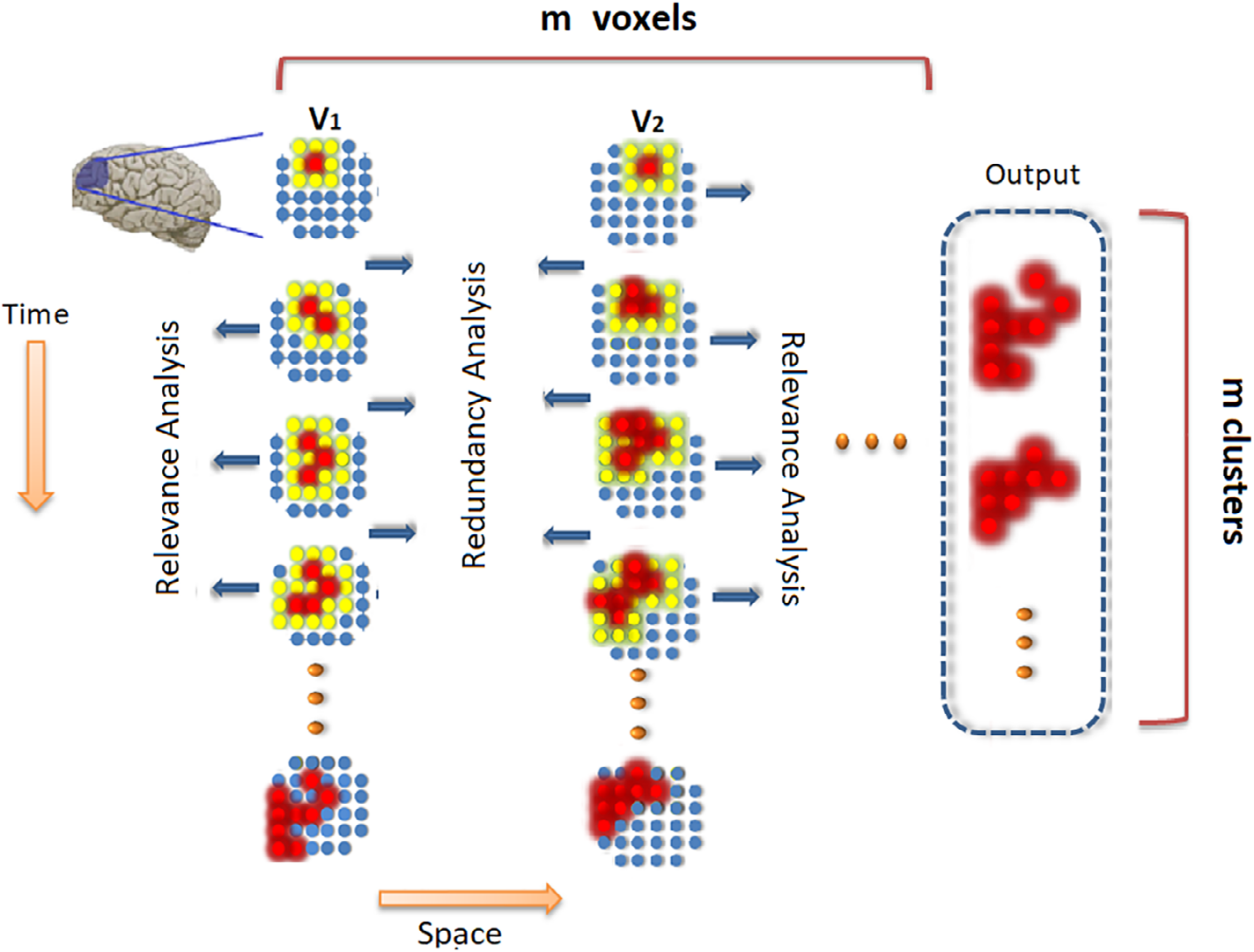
A schematic plot of an example region traversed by the proposed algorithm to detect information clusters originating from voxel *V*_1_ and *V*_2_. The algorithm first creates the information map for the cluster starting from *V*_1_ from top to bottom, and then moves to *V*_2_ and follows the same process. The red voxels are the admitted voxels and the yellow voxels represent the neighbors of the cluster at each step. The online spectral relevance analysis is performed during each search in the neighborhood layer, and the group biased mutual information redundancy analysis is performed before each next search in the neighborhood layer

As can be seen in the described steps, the cluster originating from *v*_*s*_ is expanded until no neighbors are found whose addition to the cluster enhances its discriminant score. In that case, the algorithm saves the detected cluster as well is its calculated score (information) as part of the output, and starts the same process starting from the voxel next to v_s_. The algorithm terminates when a cluster is detected starting from every voxel in the search space. A schematic plot of the steps of this procedure is illustrated in Figure 1 where the steps proceed from top to bottom (Steps 1–4) starting from every voxel. Note that the size of information clusters can span from one voxel (meaning that none of its immediate neighbors increase its information) to the entire search space (meaning that the entire search space as one cluster contains relevant information). However, both of these extreme cases were rare according to our experiments. Also, note that during Step 2, rather than selecting only one neighbor, which is the process in common greedy search methods, a group of candidate voxels of each neighborhood layer of cluster *IC* is admitted.

### 2.2 Information cluster versus activation cluster

Several statistic and algorithmic variable search methods have been suggested for areas such as gene expression (G'Sell, Wager, Chouldechova, & Tibshirani, 2016). However, these datasets do not consider the spatial characteristics of the data. Moreover, Lu, Jiang, and Zang (2003) suggested a region growing approach where clusters are located based on a homogeneity criterion and a predefined maximum cluster size determines the expansion stopping process. Also, Heller, Stanley, Yekutieli, Rubin, and Benjamini (2006) and Rosenblatt, Finos, Weeda, Solari, and Goeman (2018) propose a cluster detection method where neighboring voxels form a cluster based on the correlation of their corresponding time series. The difference between these studies and ICS is that they detect the activation clusters, that is, they aim to locate groups of contiguous voxels that are activated together and the information of the clusters do not play a role in the search process. While the contiguity of the voxels is taken into consideration in the proposed approach in this article, the voxels within the clusters might not show similar levels of activation, and instead their combination of activation levels forms the information cluster that is detected by the cluster search process. In other words, in information clusters, the activation of a cluster of voxels is not necessarily correlated. In fact, contrary to a necessarily correlating feature set, we define the conjunction of a feature set (voxel‐level activation) constitutes an information cluster, which is a principal concept in rule‐based pattern discovery.

While the proposed algorithm provides a general data‐driven framework for information detection, the proper choice of heuristic function for analysis of relevance and redundancy needs important considerations: first, it is important to take into account the interaction and structure within groups of voxels rather than considering them as merely a number of voxels placed as a group. Second, feasibility of the analysis should be considered as the number of voxels in the search space can be too large for many feature selection methods due to their time complexity. For the first point, we propose an online feature selection criterion which takes the interaction between features in to account. For the second issue, we propose an algorithmic technique for implementation of the proposed method which makes the analysis time‐efficient for experimentation on bigger search spaces such as whole brain analysis. In the next section, we describe these methodological techniques.

### 2.3 Online feature selection as heuristic function

A basic approach for evaluating the discriminant power of a group of features is the supervised feature subset selection by simply using the test accuracy of a trained machine learning algorithm. The major shortcomings of this approach include the requirement of retraining the model each time new voxels information is being evaluated as well as being model‐specific. Several other approaches have been proposed for feature set evaluation including statistical methods, linear discriminant analysis (Balakrishnama & Ganapathiraju, 1998; Welling, 2005) and spectral cluster analysis (Rousseeuw, 1987; Wilks, 2011; Zhao & Liu, 2007). The purpose of all of these approaches is to provide a measure of how separable groups of data are based on a feature set. These approaches are designed for offline feature selection where the entire feature group is known a priori. As explained previously, in the proposed ICS approach, while the samples (subjects) are constant, the features flow into the model one at a time dynamically, and are admitted to the set if they are found to be beneficial to the information of the cluster, otherwise they are rejected. As a result, we can exploit this characteristic to formulate our search procedure as a criterion known as online (or streaming) feature selection, where evaluation of the features is performed by their arrival. This formulation makes it suitable to perform dynamic feature group evaluation during the spatial search. Online feature selection is a relatively new topic for which a number of approaches have been proposed during the recent years including the gradient descent based model named Grafting (Perkins & Theiler, 2003), the likelihood ratio‐based method named Alpha‐investing (Zhou, Foster, Stine, & Ungar, 2005), and the feature redundancy and relevancy based method known as OSFS (Wu, Yu, Hefei, Wang, & Zhu, 2013). However, despite their advantages in feature selection, they do not capture the structure and correlations within the groups of attributes. Moreover, Wang et al. suggested an online group feature selection (OGFS), where the global structure of the features is considered in selecting the best subset (Wang et al., 2015). While this is a valuable quality, the use of LASSO in feature group analysis of their approach has limited capability for the domain of voxel level decoding of cognitive states due to the issues mentioned in the introduction section. We incorporate an online feature selection method inspired by OSFS and OGFS as the heuristic function of our greedy algorithm. However, we use a different process for feature redundancy analysis while considering their interaction between the voxels and group structure within the clusters. Here we explain the proposed heuristics for the suggested search approach.

### 2.4 Relevance analysis

In order to form the information clusters, informative voxels are admitted based on relevance analysis process. This can be shown as the following formula:

(1) $$\mathrm{dif}=\text{Information}\left(\mathrm{IC}\cup v_{n}\right)-\text{Information}\left(\mathrm{IC}\right)$$

In other words, if the calculated discriminant power of the addition of the newly arrived voxel *v*_*n*_ to the cluster *IC* is higher than the information of *IC* (*dif >* 0), the algorithm admits *v*_*n*_ and adds it to *IC*. As the steps of the algorithm show, this relevance analysis is performed over all of the immediate neighbors of *IC*, and the relevant subset of the neighbors joins the cluster to expand it.

As mentioned before, various feature selection methods can be applied to evaluate features individually. However, in our application, the objective is to calculate the discriminant power of group of features (feature set selection). For this purpose, we used spectral feature analysis, particularly, group‐level trace ratio of between‐class (global) to within‐class (local) affinity relationship in the data (Nie, Xiang, Jia, Zhang, & Yan, 2008). This measure ensures that samples from the same class have a higher similarity compared with samples from different classes. Therefore, if addition of a new feature (dimension) increases this separability, it is considered as an admissible feature. A main reason for using this measure is its ability in capturing the global group information within the groups of data.

The spectral feature selection attempts to find a smooth feature selector matrix based on the notions of class affiliation which measures the ratio between local and global affinity. In other words, the higher the following relation, the higher quality is the feature.

(2) $$\text{Information}\;\left(\mathrm{IC}\right)=\frac{\;\sum_{\mathrm{ij}}\|\mathrm{zi}-\mathrm{zj}{\|}^{2}\mathrm{Sb}}{\sum_{\mathrm{ij}}\|\mathrm{zi}-\mathrm{zj}{\|}^{2}\mathrm{Sw}}$$

Where with the procedure of feature selection, the data matrix *X* is transformed to *Z* by the feature space projection, *Z* = *W* ^*T*^ *X*, that is, *Z* is a transformation of *X*, and *z*_*i*_ and *z*_*j*_ are the corresponding values of *z* for data points *i* and *j*. Also, *S*_*b*_ and *S*_*w*_ denote the Fisher scores of between and within class adjacency matrices (Nie et al., 2008) which are calculated as follows:

$$\;\left(\mathrm{Sb}\right)\mathrm{ij}=\left\{\begin{array}{c} \frac{1}{n}-\frac{1}{n_{l}},\text{if}\;i\;\text{and}\;j\;\text{belong to the same class}\;\\ \frac{1}{n},\text{Otherwise} \end{array}\right.$$

$$\left(\mathrm{Sw}\right)\mathrm{ij}=\left\{\begin{array}{c} \frac{1}{n_{l}},\text{if}\;i\;\text{and}\;j\;\text{belong to the same class}\;\\ 0,\text{Otherwise} \end{array}\right.$$

where *n*_*l*_ denotes the number of data points from class *l*. We can also consider *S*_*b*_ as the adjacency matrix of graph *G*_*b*_, and *S*_*w*_ as the adjacency matrix of graph *G*_*w*_ which represent between‐class (global), and within‐class (local) affinity relationship among the data points, respectively. In other words, in both graphs *G*_*b*_ and *G*_*w*_, the nodes are the subjects, and the edges correspond to their class affiliation. Fisher score is a supervised method therefore makes use of the label information for constructing the weight matrices *S*_*b*_ and *S*_*w*_. When the label information is not available, Laplacian score can be applied for constructing the two weight matrices instead.

The degree matrix *D*_*w*_ of the graph *G*_*w*_ (within‐class) can be defined as *D*_*w*_ = *diag* (*S*_*w*_) if *i* = *j*, and 0 otherwise, and its Laplacian matrix can be defined as *L*_*w*_ = *D*_*w*_ − *S*_*w*_ (Mohar, Alavi, Chartrand, Oellermann, & Schwenk, 1991; Wang et al., 2015). Similarly, the Laplacian matrix of graph *G*_*b*_ (between‐class) is defined as *L*_*b*_ = *D*_*b*_ − *S*_*b*_.

With the property of Laplacian matrix, for a feature subset(cluster) *IC*, we can obtain the following equivalence from Equation (2):

(5) $${\left\|z_{i}-z_{j}\right\|}^{2}S_{b}=W_{I}C^{T}\left({\mathrm{XL}}_{b}X^{T}\right)W_{\mathrm{IC}}$$

Therefore, Equation (2) can be converted into the trace ratio of the form below (Nie et al., 2008; Wang et al., 2015):

(6) $$S\left(\mathrm{IC}\right)=\frac{\mathrm{tr}\left(W_{I}C^{T}\left(XL_{b}X^{T}\right)W_{\mathrm{IC}}\right)}{\mathrm{tr}\left(W_{I}C^{T}\left(XL_{w}X^{T}\right)W_{\mathrm{IC}}\right)}$$

And the spectral quality for the arriving feature *v*_*i*_ can be measured by a feature‐level spectral score defined below:

(7) $$S\left(v_{i}\right)=\frac{w_{i}^{T}\left(XL_{b}X^{T}\right)w_{i}}{w_{i}^{T}\left(XL_{w}X^{T}\right)\mathrm{wi}}$$

Therefore, given the selected feature subset *IC*, the new arriving feature *v*_*n*_ will be admitted if its inclusion improves the discriminative ability of the feature subset (calculated via Equation (6)), that is Equation (1).

### 2.5 Redundancy analysis

By performing an intragroup pruning before further expanding the cluster *IC* ∪ *v*_*n*_, the optimal group of voxels *V*_opt_ ∈ {*IC* ∪ *v*_*n*_} can be selected. This process further increases the precision of the information map by removing residual redundancies as a high quality set of features should not only be individually relevant, but also should not be redundant with respect to each other.

For this analysis, we consider the interaction between the voxels within the clusters to be able to capture the complex structures among groups of voxels rather than only assessing their individual predictive power. Therefore, the redundancy score of each feature is calculated as its mutual information with the rest of the features in the group which is formulated below (Brown, 2009).

(8) $$J=\frac{1}{n-1}\sum_{k=1}^{n-1}I\left(X_{n};X_{k}\right)$$

where *I* denotes the mutual information between two vectors, *X* is the set of features, and *n* is the number of features. A large value of *J* represents high redundancy, that is, low quality of the feature. Also, the mutual information *I* is calculated by the following formula:

(9) $$\mathrm{MI}=\sum_{x\in X}\sum_{z\in Z}P\left(x,z\right)\log\left(\frac{P\left(x,z\right)}{P\left(x\right)P\left(z\right)}\right)$$

where *x* denotes the feature values and *z* represents the class labels.

### 2.6 An efficient implementation

As discussed in the previous section, the proposed approach performs a set of calculations in every step of the search to investigate every voxel at the vicinity of the detected feature clusters. Therefore, a large fraction of the calculations is repeated due to the overlap among regions, that is, a voxel can be visited multiple times. This significantly affects the analysis time and can make search over large regions impractical. We apply this improvement by breaking down the calculations for every voxel and storing them at hash tables to be later used in the search process. At every step of the search, the algorithm first checks if the fragment of calculation encountered during the search already exists in the tables. If so, it directly uses the previously calculated values recursively to save time; otherwise, it performs the necessary calculation and stores it in the table for future use. This top‐down approach significantly increases the search time, making precise information detection on voxel‐level resolutions over large search spaces possible. Time complexity of ICS based on our experimentation setup is discussed in the discussion section.

For relevance process, based on Equation (7), the majority of computational burden is on the matrix multiplication *XL*_*b*_ *X* ^*T*^. However, we can make the above matrix multiplication only once, before starting the search, and preserve the values inside a hash table instead of recalculating it during search. Due to the property of trace of matrices, the value for each voxel can be easily obtained by looking up the precalculated values in the hash table.

**Figure 2:**
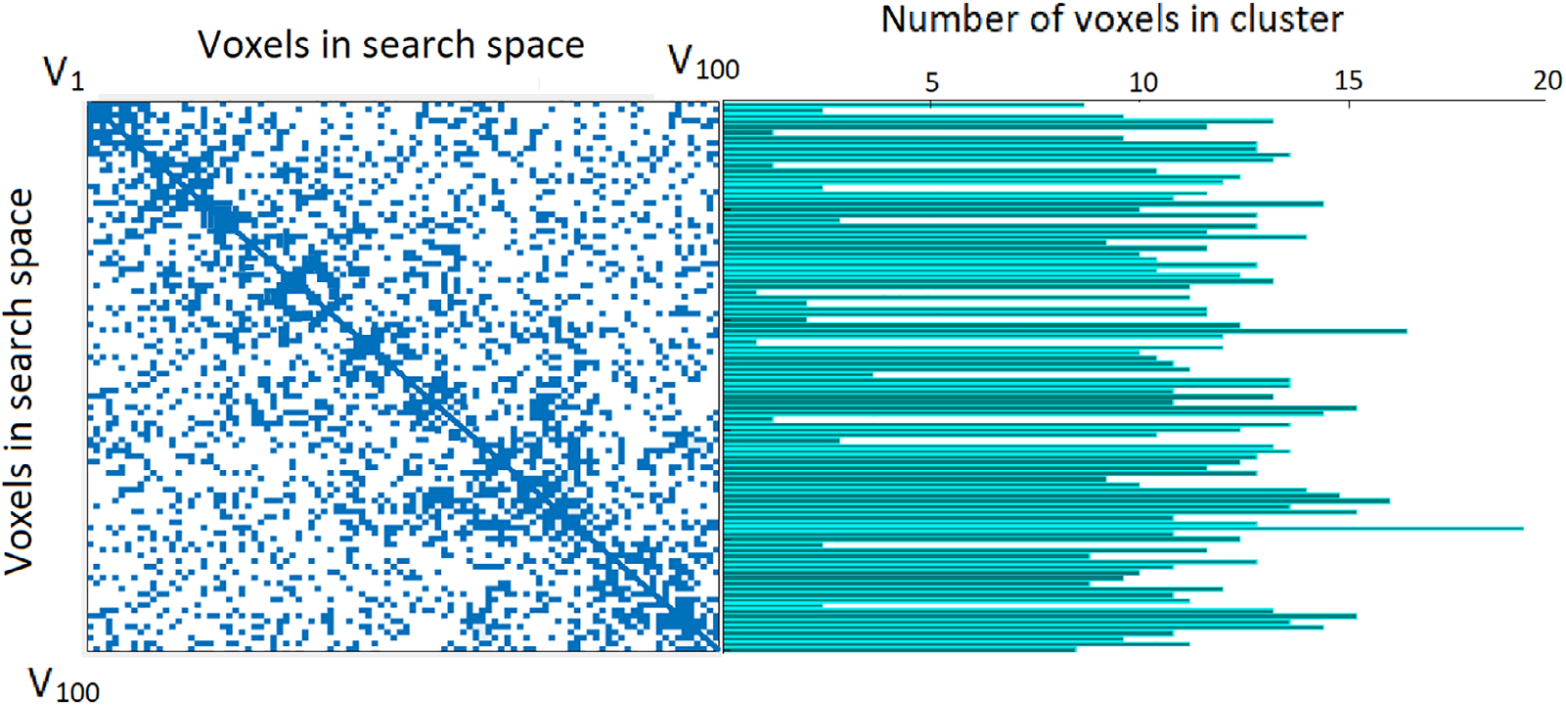
An interaction matrix after a complete search over 100 voxels. Nonzero elements are depicted in blue color. The right figure illustrates the size of clusters originating from each voxel

Moreover, for redundancy analysis, we can break down the calculations of each voxel in Equation (8). This is due to the fact that the mutual interaction information between each voxel and every other voxel is a sum calculation, and the individual value of each feature can be looked up after being calculated and stored once. Therefore, each time redundancy analysis is being performed on a feature set *IC*, for each voxel, the algorithm first checks if the calculation for it exists in the hash matrices, and only performs the calculations and saves them if they have not been performed previously. This memoization process is included in the pseudocode in the Supplementary Information. Note that the calculations for eight based on this approach are only performed when needed, which is more efficient than precalculating the interactions for each voxel and every other voxel. This is due to the fact that we are interested in detecting redundancies within an analytically formed cluster of voxels which only requires the interaction of voxel being visited with the members of that cluster rather than its interaction with every other voxel in the entire search space. As a result, the mutual information matrix becomes rather sparse. An example of interaction matrix on 100 voxels in Autism Brain Imaging Data Exchange (ABIDE) dataset is depicted in Figure 2 where each element shows the mutual information between two voxels in the search space, and nonzero elements are shown in blue color. As can be seen in that figure, many cells are empty, meaning that the search did not require calculation of the interaction between those pairs of voxels. As we can also observe in that matrix, the density of nonzero elements is higher in the vicinity of the diagonal which indicates the locality of search in the vicinity of functional volumes. The size of clusters originating from each voxel is also illustrated on the right side of Figure 2. Note that the cluster sizes do not necessarily match the number of nonzero (blue) cells in each row of the interaction matrix. This is due to the pruning step that the algorithm performs, which separates the informative subset of voxels within the cluster during each redundancy analysis step.

### 2.7 Interpretation of the output

**Figure 3:**
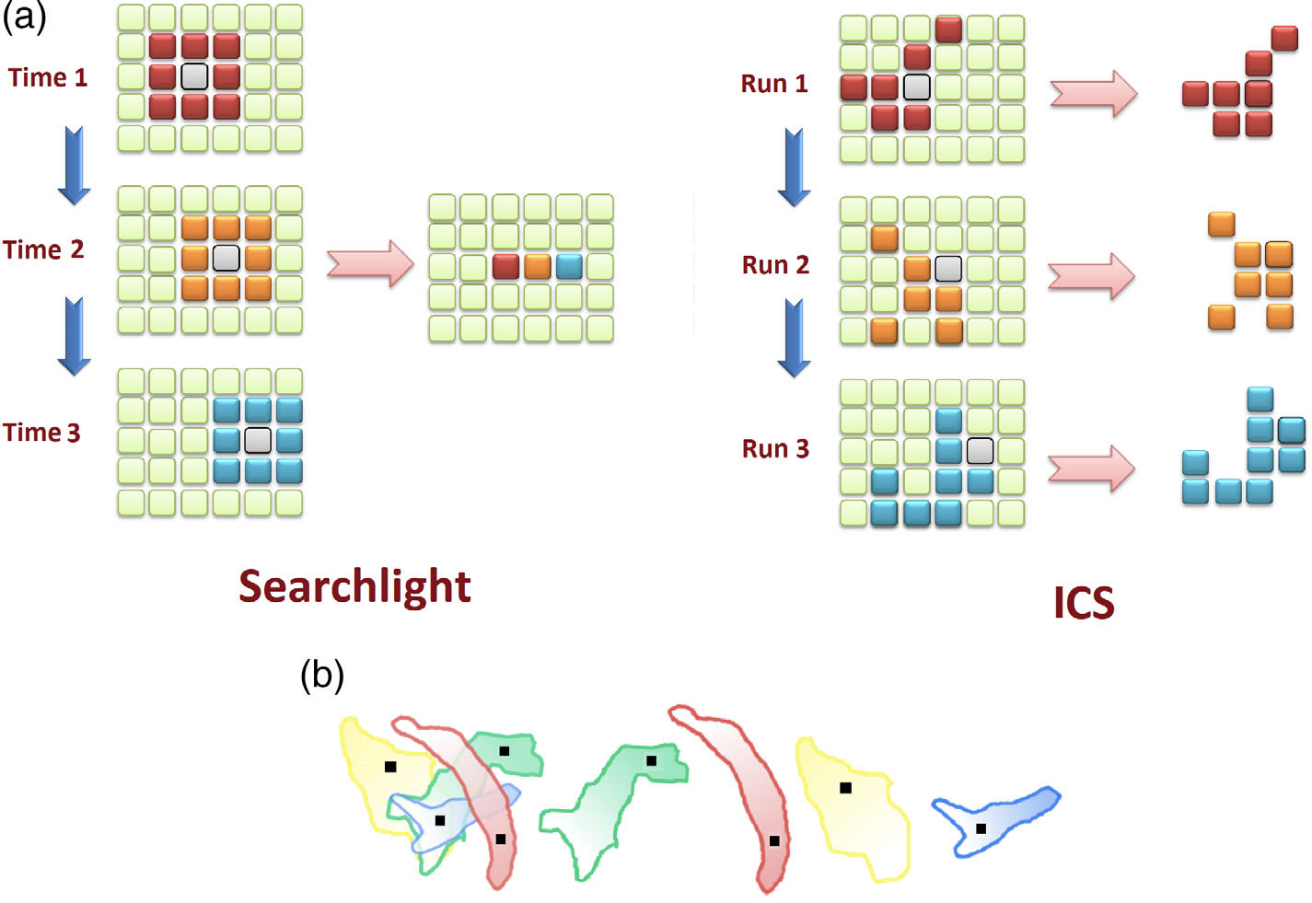
(a) An illustration of the steps and output of searchlight procedure compared with the ICS algorithm. The gray voxel is the search sphere center voxel in the searchlight method, and the starting voxel in ICS algorithm. The radius for searchlight in this schematic illustration is one voxel, and the information of the searchlight sphere, denoted by a specific color for each sphere is assigned to the voxel at the center of the sphere, that is, each voxel in the output map has the same color (information) as its search sphere. On the other hand, the output of the ICS method is a set of clusters expanded from the starting voxel through a data‐driven heuristic. The information of each cluster is demonstrated by a specific color. (b) Left: An example illustration of overlapping clusters created by ICS. Right: The same clusters depicted individually. The voxel indicated by black dots are the starting voxel *v*_*s*_ which are expanded based on the discriminant analysis heuristic, resulting in a specific discriminant score for each cluster

The direction of search based on ICS algorithm is guided only by the underlying information in the search space. This means that emergence of overlapping clusters with different starting points is a possibility in the proposed schema. Therefore, the output of ICS can be presented as a set of clusters with their measured discriminant scores. Figure 3 presents a comparison between the outputs of the searchlight procedure with the ICS algorithm after three steps of each method. As can be seen in that figure which is depicted in two dimensions for suitable demonstration, the output of the searchlight approach is a map where the value of each voxel represents the information of the searchlight surrounding it while the output of ICS is a set of clusters originated from each voxel. In other words, the information of a cluster in ICS process is automatically assigned to the entire cluster. The same figure also shows how clusters originating from different voxels can overlap one another in the ICS schema. The separated clusters on the right show a representation of the actual output where each cluster bears a certain information value. Therefore, each cluster is assigned an information score, and each voxel can be associated with multiple clusters as various combinations of features bear different predictive power. From a predictive modeling point of view, each information cluster can be considered as a feature whose information value represents its classification quality. Therefore, a cluster with high information value is more suitable for prediction and diagnosis purposes.

## 3 EXPERIMENTAL RESULTS

### 3.1 Experimental setup and data

The source code of the proposed method is available at https://github.com/ThisIsNima/ICS. All the experiments were performed on an Intel Core i7‐3370 CPU, 3.40 GHz with 32 GB of RAM.

In order to evaluate the proposed approach, we first derived the information map of the fMRI datasets of two case studies based on the suggested algorithm as well as the searchlight and LASSO within generalized linear model procedures as the base line, and then selected the clusters which provide above chance (bigger than 50%) information for one setting, and their top 50 clusters for another experimental setting as the classification features. Fivefold cross‐validation was performed during the feature‐selection phase for each of the three approaches. Then, we compared the area under the curve (AUC) of a classifier which was trained on the three generated feature vectors to assess the quality of the generated features on the same dataset. For this purpose, we create two case studies using real fMRI data as well as a synthetic dataset.

The first case study includes a real fMRI dataset of 683 subjects from the publicly available ABIDE database (Di Martino et al., 2014). This worldwide multisite database includes resting state fMRI images of 370 healthy subjects, and 313 subjects diagnosed with Autism spectrum disorder (ASD). Also, despite the variances existing in this dataset due to diversity of data sources, we performed the analysis on the subjects as one group of data. Previously preprocessed rs‐fMRI data were downloaded from the ABIDE database. This dataset was selected from the C‐PAC preprocessing pipeline. The fMRI data were slice time corrected, motion corrected, and the voxel intensity was normalized using global signal regression. As mentioned in Section 2, the input to the algorithm is the set of activation time courses of every voxel averaged over time, that is, and *M* × *N* matrix, where *M* is the number of subjects and *N* is the number of voxels in the search space.

To present the experimental results, we first compare the whole brain analysis performance, and for further investigation, we assign the search space to be regions of interest which are widely believed to play a crucial roles in ASD, namely the hippocampus, amygdala, and cerebellums (Baron‐Cohen et al., 2000; Bauman & Kemper, 1985; Fatemi et al., 2012; Schumann et al., 2004). The automated anatomical labeling (AAL) atlas was used to extract the regions of interest.

The second case study included simulated data where time courses of an average fMRI data with two conditions were generated for a population of 1,000 subjects based on values extracted independently from a Gaussian distribution for four different sizes of search spaces of size 100 voxels; 500 voxels; 10,000 voxels; and 30,000 voxels. Also, noisy values were added to the signal with the constraints of realistic degree of correlation between adjacent voxels. A spatial pattern of response was then introduced to the two conditions which faded in and out according to the average temporal pattern of the cardiovascular response pattern among adults.

### 3.2 Prediction results

The prediction results of the proposed approach are provided in Figures 6 and 7 where the area under the receiver operating characteristic curve is used as the evaluation measure.

After the discriminant scores of the clusters are revealed, the clusters with above chance information and the top 50 clusters were used for classification in two settings. Note that a cluster was created based on the ICS approach, the LASSO regularization method, and the searchlight process for each voxel where in ICS, the cluster was expanded from each voxel *v*_*i*_, and for the searchlight procedure, it was the searchlight that encompassed each voxel *v*_*i*_ by a certain radius, and for LASSO, it is the single voxels that are found to be significant in relation to the dataset labels. Similar spectral discriminant score of the relevance analysis in ICS was used as the analytical measure for the searchlight approach to make an appropriate comparison between the two approaches. In other words, the spectral discriminant score of each group of voxels surrounded by a search sphere was assigned to the voxel at the center of it. Based on both methods, each cluster contains an information value. The data were split in to train and test segments where 80% of the data were used for training, and 20% for testing. The information clusters were extracted from the training set. For the first setup, clusters with over 50% discriminability power were selected from the outputs of all three methods, and for the second setup, top 50 clusters (voxels in case of LASSO) constituted the feature vectors, thus generating three feature sets for each of the two settings. Then, an SVM model was trained based on each of the feature sets, and was used to predict the labels of the test data. The reason for using a classification model (SVM) different than the information mapping models is to validate the generalizability of the analysis.

#### 3.2.1 Full brain analysis results

**Figure 4:**
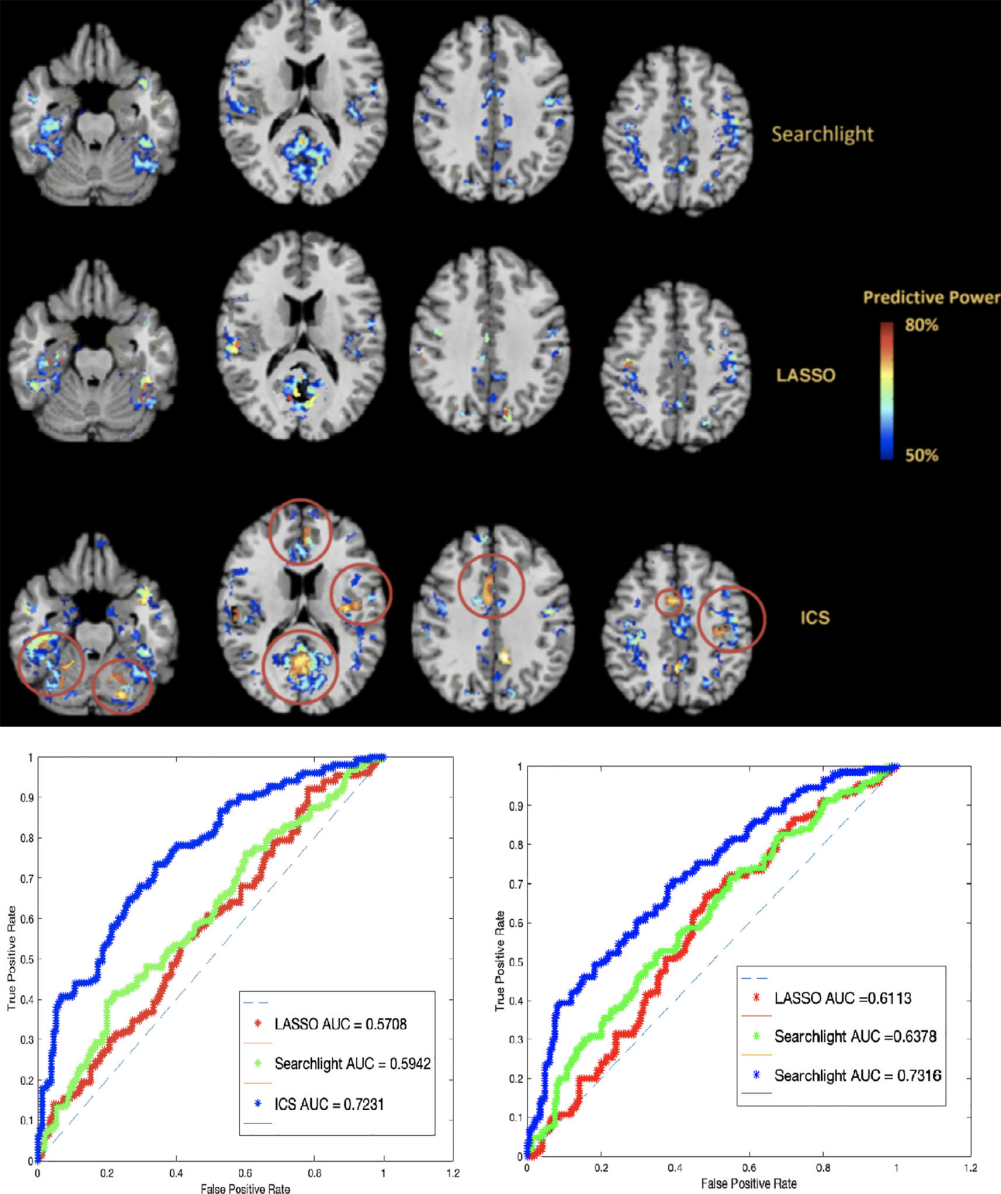
Top: A comparison of the above chance accuracy clusters derived using the searchlight process (top row), least absolute shrinkage and selection operator (LASSO) (middle row), and the ICS algorithm (bottom row) on the Autism Brain Imaging Data Exchange (ABIDE) data set. Major differences between the three maps are indicated by the red circles. In case of overlapping clusters generated by ICS, the clusters with the highest predictability were selected for this visualization. Bottom‐left: Classification performance on full‐brain search space for ABIDE dataset based on the above chance clusters as the features. Bottom‐right: Classification performance on full‐brain search space for ABIDE dataset based on the top 50 clusters as the features. The train‐test population for both settings was 546–137, respectively

A visualization of the informative clusters (above 50% information) detected by ICS as well as the searchlight approach and LASSO regularization is presented in Figure 4. As can be seen in that figure, the above chance clusters presented by both the searchlight procedure and LASSO regularization show a lower discriminant score (maximum: 57.7%, average: 53.5% for searchlight, and maximum: 61.3%, average: 52.2% for LASSO) compared to the ICS clusters (maximum: 73.1%, average: 69.8%). Also, while there are a number of regions where all three approaches detect similar informative areas, ICS detects an arrangement of voxels with a group structure which shows an increase in the information. These regions include the posterior cingulate cortex, Wernicke's area, the amygdala, and left insula. Furthermore, ICS detected clusters with above chance information that the compared approaches were not able to detect (their detected information were below 50%). These regions include portions of the cerebellum, and the anterior cingulate cortex (ACC). These results are in line with a number of previous studies on ASD.

For example, the role of abnormalities in cerebellum from both an information mapping point of view and cortico‐cerebellar functional connectivity in detection of ASD has been demonstrated in several studies (Ramos, Balardin, Sato, & Fujita, 2018; Traut et al., 2018). Moreover, Cascio et al. found that the ASD adults were underresponsive to the pleasant and neutral tactile stimuli while showed greater activation in the posterior cingulate and insula in response to the unpleasant tactile stimuli (Cascio et al., 2012). Furthermore, numerous studies demonstrate that abnormalities in activation patterns and functional connectivity in the insula and ACC contribute to ASD (Zhou, Shi, Cui, Wang, & Luo, 2016). Therefore, the results of this analysis further demonstrates that from an information mapping point of view, the activation patterns of the mentioned areas can prove more valuable in detection of ASD.

Classification AUC of the three approaches using both the above chance features as well as the top 50 features were also measured after an SVM model was trained based on the three feature vectors for each setting separately. The search space for this analysis is the entire brain. For the searchlight method, radius values from 1 to 10 voxels were examined, and the highest performance, which belonged to the search sphere of three voxels, was selected. For LASSO, the *λ* value was set to 0.001. As can be seen in that plot, the classifier trained on the ICS features on both settings significantly outperforms the classifier trained on the searchlight and LASSO features.

The precision of the three information decoding approaches on synthetic data is provided in Figure 10 where the generated datasets contain 100; 500; 10,000; and 30,000 voxels. Similar to the real dataset, fivefold train‐test validation was also arranged for this set of experiments. Therefore, the data for 800 subjects were used for information mapping, and the remaining 200 subjects were used for classification test based on the top 50 informative features according to the analysis on the training set. As can be seen in Figure 10, the ICS method improves the classification accuracy over the compared approaches in all four setups.

#### 3.2.2 Cluster level analysis results

**Figure 5:**
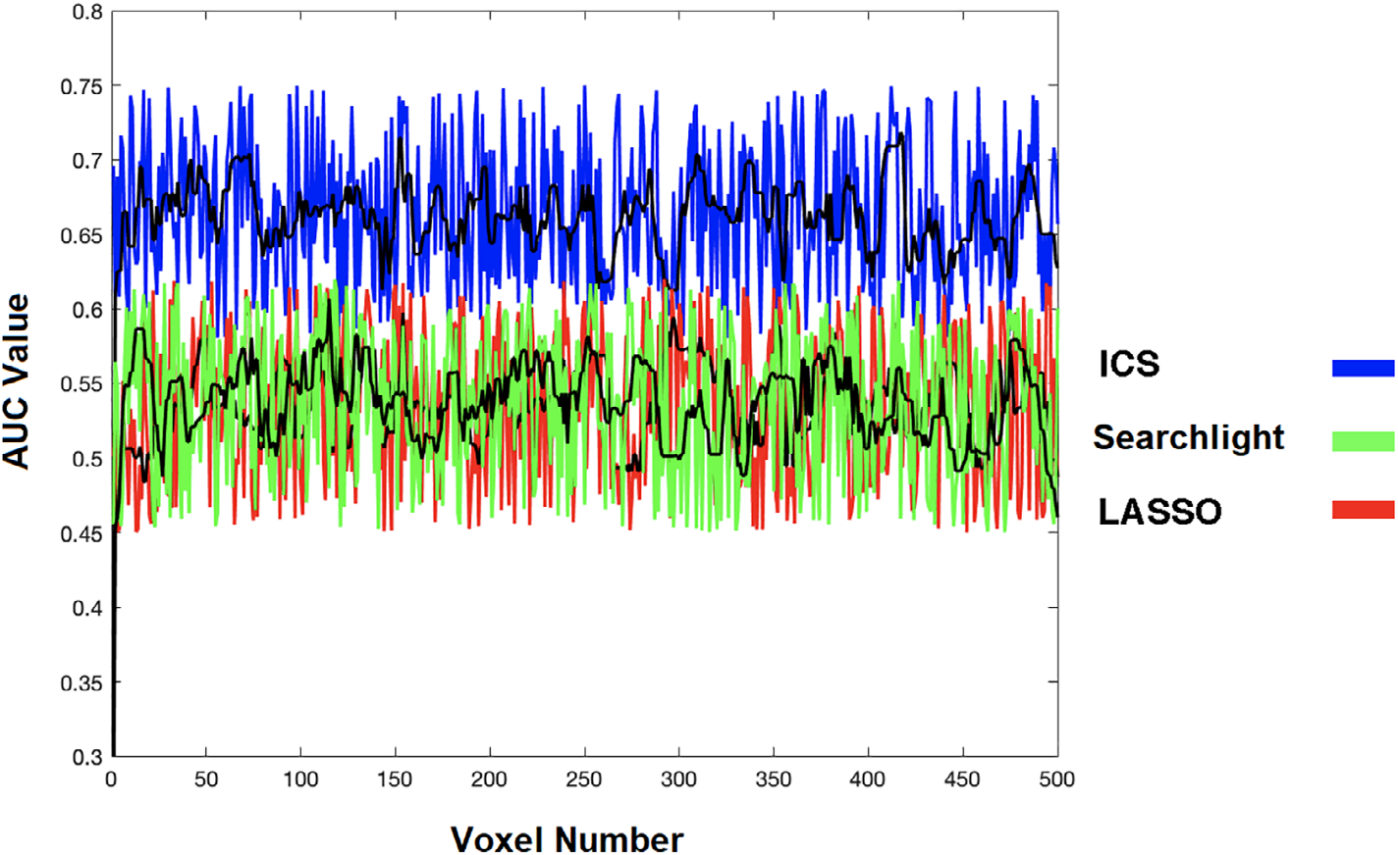
Comparison of classification area under the curve (AUC) with SVM between ICS, least absolute shrinkage and selection operator (LASSO), and searchlight with voxel radius set to 3 for left Crus II of the cerebellum (region 93 per automated anatomical labeling [AAL]) with 573 voxels

**Figure 6:**
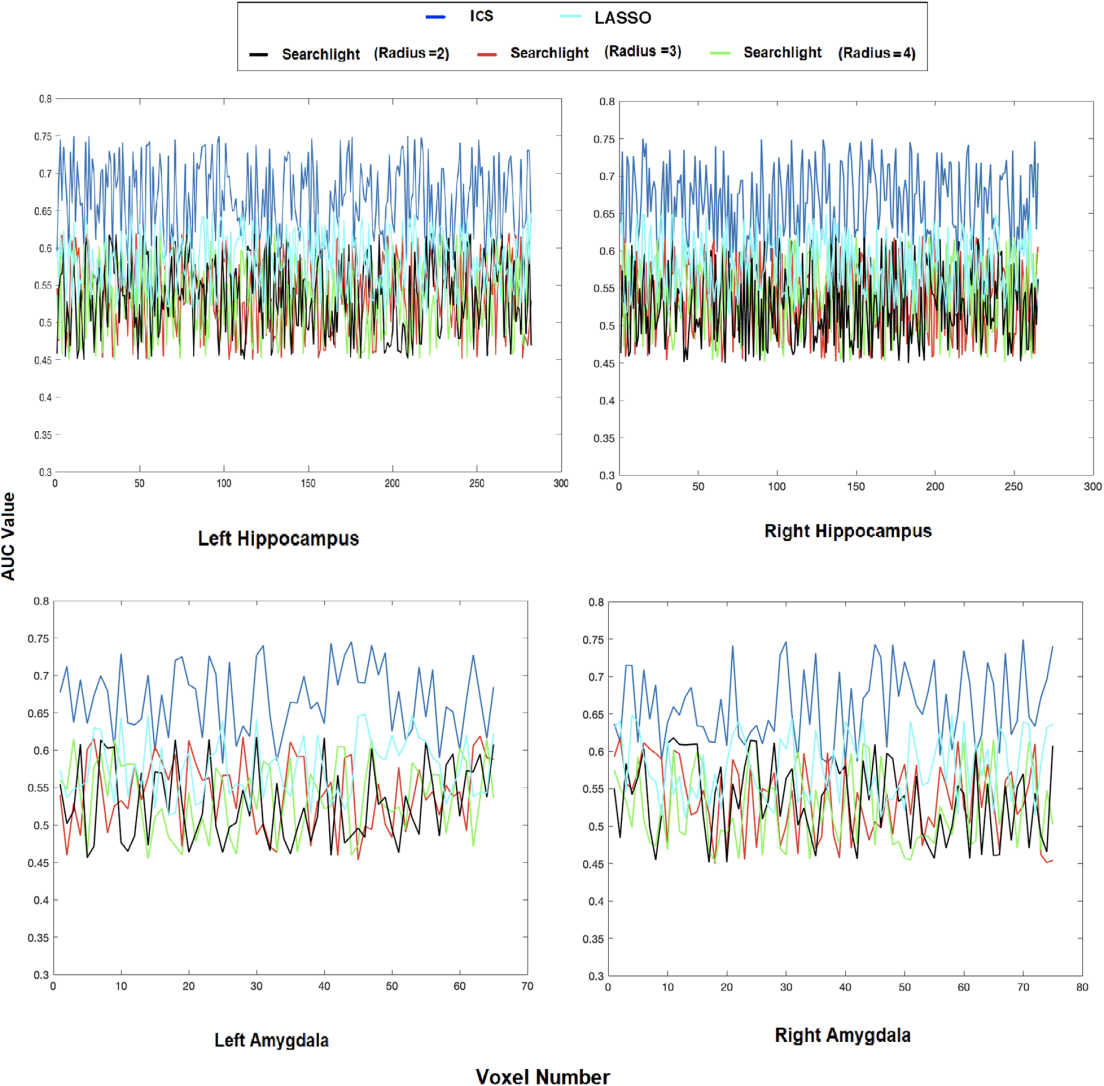
Test area under the curve (AUC) for classification with SVM of the ICS algorithm, least absolute shrinkage and selection operator (LASSO)‐based features, and the searchlight method with different search radii for right and left hippocampus and amygdala from the Autism Brain Imaging Data Exchange (ABIDE) dataset

**Figure 7:**
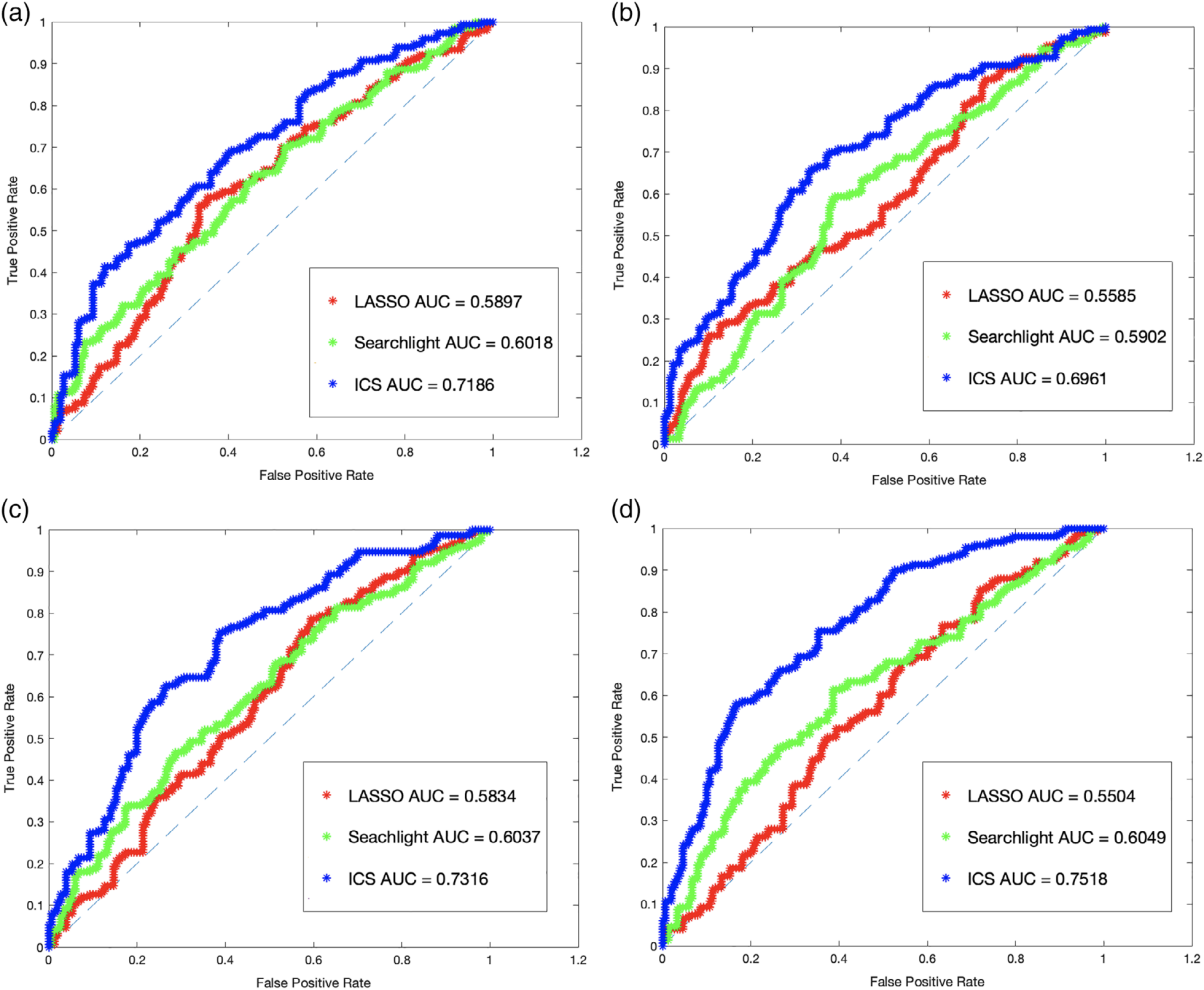
Area under the receiver operating characteristic (ROC) curve for synthetic datasets of size 100 (a); 500 (b); 10,000 (c); and 30,000 (c) voxels based on the top 50 features according to the three approaches

The classification performance of the three approaches can also be assessed in smaller search spaces with more specific topological properties. This analysis is important due to the fact that assignment of a fixed searchlight radius on large search spaces might guarantee excellent performance in specific regions while underperform in other regions. In Figure 5, the classification accuracies based on every feature within the left Crus II of the cerebellum (Region 93 per AAL) are compared among the three methodologies as an example. Analytical results of more regions are provided in the Supplementary Information. In that figure, the AUC value assigned to every voxel for the searchlight method is the calculated AUC that its search neighborhood with three voxel radius provides, that is, the information of the individual clusters (searchlights). For ICS, this value for every voxel corresponds to the measured AUC for the cluster originated from it, and for LASSO, the feature vector contains the voxels found to have the highest predictive powers. Five runs of analysis based on both methods are presented in that figure, and due to the fluctuations in the AUC values as a result of random selection of the train‐test samples, the average AUCs are indicated by the black line. As can be seen in that figure, ICS consistently demonstrates superiority in terms of classification accuracy compared to the other two approaches. More comparisons of the three mapping methods on specific regions of interest are provided in Figures 6 and 7, which further displays improvement of information map accuracy from the ICS method.

The experimental examinations of the proposed information mapping algorithm shows a significant enhancement over the searchlight procedure over both real and synthetic datasets. The higher classification test accuracy of ICS points to the data‐driven advantage of identifying the informative combination of voxels that form various informative clusters. This is because besides considering the combination of the voxels in their proximity, the formation structure of the groups of voxels as well as their interaction with one another also play important role in the information they provide. Moreover, redundant voxels are dynamically removed by ICS, which contributes to further enhance the precision of the discovery. These qualities are assessed through the online spectral feature evaluation and the spatially biased mutual information procedure. In other words, the ICS increases the information cluster dynamically as it searches for informative voxels to recruit for expanding the cluster while reassessing the MB in the existing feature set and removing the redundancies. While high precision is a crucial quality for information mapping of the brain, the proposed procedure benefits from other advantageous characteristics which are discussed in the next section.

### 3.3 Detection of simulated effects

**Figure 8:**
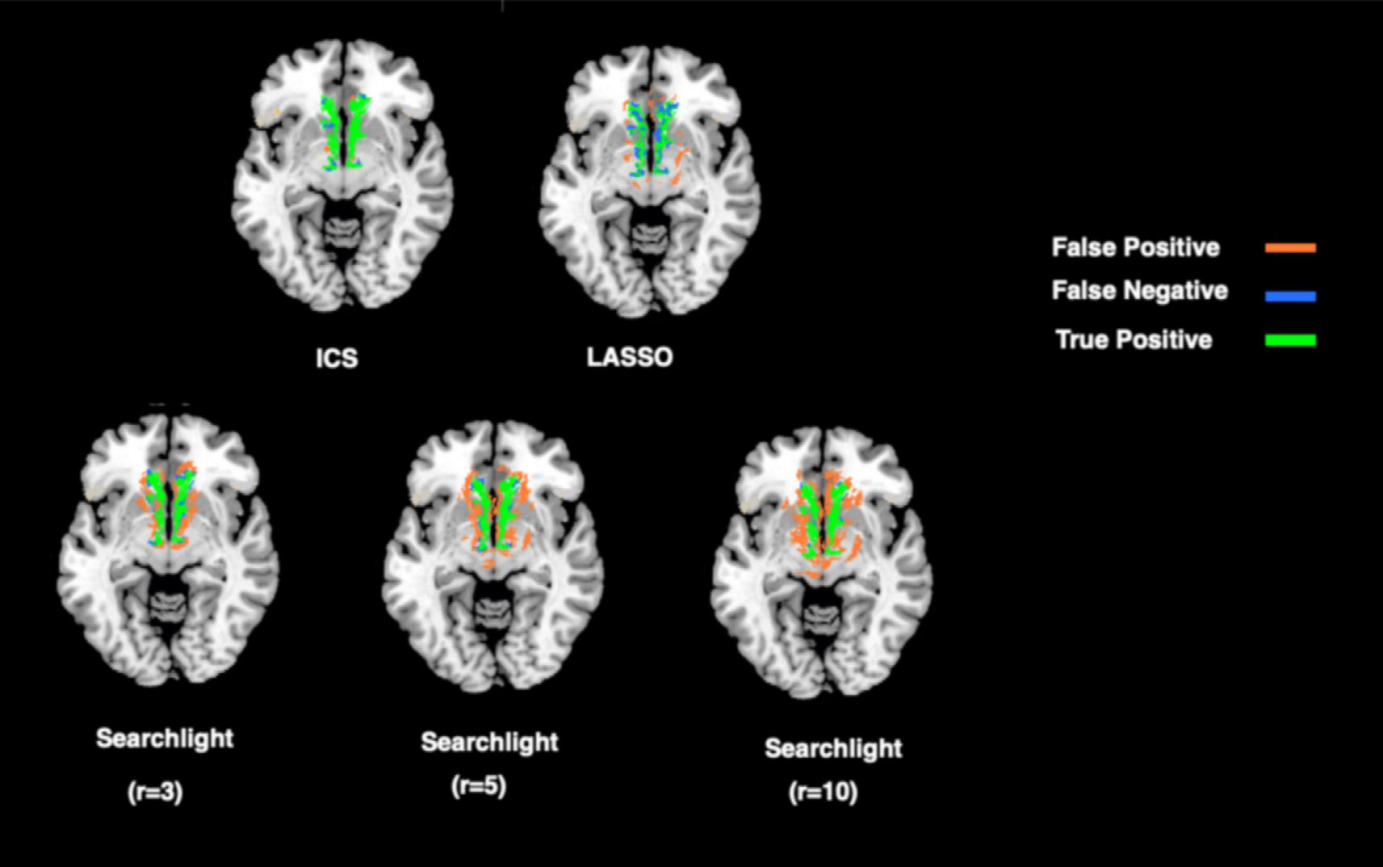
Detection results obtained by all methods using the dataset with 25% simulated atrophy

In order to assess the sensitivity of the proposed method in detecting the underlying effect of interest, we tested it in a simulated setting where 25% atrophy where imposed on the frontal lobe mask. Then, we compared the three methods to the ground‐truth by mapping their true and false positive rates and well as false negative detection rates. The result of this analysis is depicted in Figure 8. As can be seen in that figure, the rate of false positive in the searchlight approach is higher than both ICS and LASSO‐based approach while the false negative rate in LASSO is higher compared to ICS and searchlight. This can be explained by the fact that, as mentioned in Section 1, the searchlight method tends to declare a subregion with a few highly informative voxels as highly informative. On the other hand, the higher rate of false negatives in LASSO can be explained by its tendency to punish the features with smaller significance. Moreover, LASSO does not take the local characteristics of the voxels and their neighborhood into account, which can further skew its selection of the informative voxels.

#### 3.3.1 Spatial specificity

**Figure 9:**
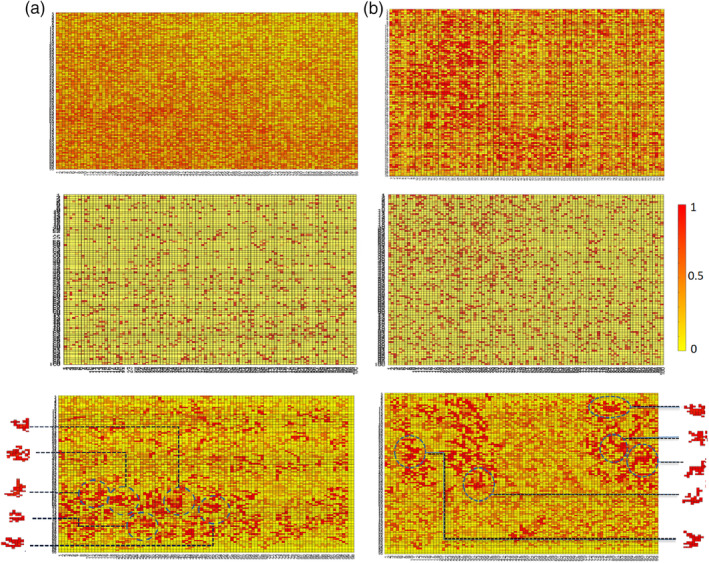
Information maps created through searchlight (top row), least absolute shrinkage and selection operator (LASSO) (middle row) and ICS (bottom row) approaches. (a) The voxel‐level map of left Crus II of the cerebellum (Region 93 per automated anatomical labeling (AAL)) and (b) belongs to Cerebellum 4 (Region 97 per AAL). For both regions, two dimensional subsegments (100 by 100 voxels) of the information maps are depicted in this figure to facilitate readable illustrations. Moreover, five sample information clusters derived from these maps through the ICS approach are depicted on the sides of the maps. In case of overlaps, the information clusters with higher quality (darker red) overshadow the ones with lower information

Spatial specificity can be defined as the ability to extract independent information from two separate regions in close proximity. As explained in Section 2, ICS detects groups of voxels whose combined activation results in an information cluster that is distinguishable between two or more groups of subjects. Therefore, each such group of voxels is assigned a unique information value. This process is in contrast with the searchlight information assignment where the information of a neighborhood with predefined size is assigned to the voxel at the center of it. Figure 9 illustrates information maps generated through searchlight and ICS approach for two regions corresponding to the cerebellum. In case of overlapping information clusters in ICS, the segments with higher information overlay regions with lower information to facilitate readability of the map. As mentioned previously, the information detection and assignment procedure of ICS offers a higher specificity, which can be observed in Figure 9.

## 4 DISCUSSION

We suggested a new MVPA method with the objective of increasing the precision of the voxel‐level information map while eliminating several constraints including the requirement for parameter tuning. We proposed a data‐driven framework which performs a search based on a data‐driven heuristic function, and starting from each voxel, dynamically detects the appropriate formation of its combination with other voxels in its vicinity.

Since ICS is able to pick up clusters with irregular shapes (and each voxels performance is not affected by its surrounding neighbors), when combined with high‐resolution scans, it can be used to: (a) associate function with its underlying fine‐grained structures such as sulcal and gyral morphology (i.e., the irregular shapes of functional maps can be compared with structural T1 images to link morphological and functional variation); (b) accurately detect the functional boundary across representations or tasks/conditions and compare them with other boundary mapping approaches; and (c) provide insights for some fundamental but unresolved brain physiological questions such as whether information in the brain is represented in a continuous or discrete form. The traditional searchlight method mixes and merges neighboring voxels information and thus is inherently biased for a continuous gradient‐like representation form, but ICS is not limited to such bias (Tee & Taylor, 2018).

### 4.1 Information of voxel clusters is discovered precisely

Due to the data‐driven expansion of clusters around each starting voxel, their calculated information is automatically assigned to the individual clusters without redundant voxels. Therefore, exaggeration of the spatial boundaries of informative areas is avoided. This property removes the possibility of discontinuous detection, that is, existence of one or few highly informative voxels dominating the information of a region which increases the possibility of overfitting.

### 4.2 Minimal parameter tuning is required

The elimination of tuning parameters such as searchlight shape (spherical or cubical) and radius, or machine learning hyper parameters (example: deep learning‐based methods) or regularization parameters (the *λ* value in LASSO or elastic net) not only increases the generalizability of the results, but also increases the efficiency of analysis. The latter point is due to the fact that the requirement for multiple runs of the analysis with different searchlight radii and then selecting the highest performing parameters is removed. Moreover, this property of the ICS algorithm resolves the issue of heterogeneous accuracy on various regions which normally occurs due to assignment of a single searchlight radius for larger brain regions with various topological characteristics. Plus, parameter tuning requires researchers to have advanced expertise of the methods they intend to use, especially with regards to manifold learning.

### 4.3 The shape of the clusters is not bound by any constraints

The shape of the searchlight sphere can affect the information detection precision. For example, in the presence of an elliptical cluster, a spherical searchlight could fail to detect its complete boundaries, thus creating an imprecise information map. However, the shape of the clusters created by ICS merely depends on the information of the voxels and their combination with the voxels in their vicinity. This can specifically be a useful property for clusters located at the edges of the search space that are more prone to irregular shapes.

### 4.4 Prediction accuracy is enhanced simultaneous to cluster detection

The search space traversal of ICS is navigated toward voxels that increase the information of the clusters as its objective is to solve the optimization function that finds the combination of neighboring voxels that maximize the information. Therefore, starting from each voxel, the search continues as long as possibility exists for enhancing the discrimination power of the cluster by adding useful voxels.

### 4.5 ICS is applicable to both supervised and unsupervised settings

Due to the spectral discriminant analysis incorporated in ICS, it is robust to problem settings with regards to labeled or unlabeled datasets. As explained in Section 2, switching to the unsupervised problem setting can be performed during construction of the weight matrices. In other words, Fisher score can be used to take advantage of label information for constructing the weight matrices while Laplacian score can be leveraged for constructing the two weight matrices when no label information is presented. This characteristic further increases the adaptability of ICS to different problem settings over other group level MVPA approaches.

### 4.6 Additional analysis is not required for interpretation of the information map

Due to data‐driven assignment of discriminant scores on the voxel level, clusters with clear boundaries are generated by the ICS method. Therefore, unlike some of the other MVPA methods, complementary tests are not required to detect informative voxels within the clusters. Moreover, the proposed approach provides higher intuitiveness compared to methodologies often considered as “black box” such as deep learning‐based approaches or regularization‐based methods.

### 4.7 State‐of‐the‐art feature set analysis approaches can be incorporated in ICS

While we employed spectral feature set analysis for the expansion step (relevance analysis) and the trace ratio procedure for the pruning step (redundancy analysis), other state‐of‐the‐art approaches can be incorporated in the ICS online search schema. Therefore, within the same spatial feature cluster search algorithm, other feature set analysis process can be applied, which indicates the flexibility of this approach.

### 4.8 Global optimality discussion

The conventional step‐wise greedy search method for feature selection yields suboptimal feature subsets due to falling in local minima (Vafaie & Imam, 1994). This is due to the fact that the choice of features depends on the order of their selection. However, in the approach proposed in this article, for every starting voxel, multiple useful neighbors are admitted at each neighborhood layer (Step 2 of the algorithm), therefore reducing the chance of falling in local optima. Moreover, the output of the algorithm includes the discovered information clusters starting from every voxel in the search space. In other words, Steps 1–4 are performed for every voxel in the search space, resulting in a more thorough search over possible voxel combinations. While this algorithm does not perform an exhaustive search over every possible combination of voxels, these two properties significantly decrease its chance of falling into local optima. In addition, global information within clusters is taken into account during both spectral relevance and redundancy analysis. Note that the problem of finding the best set of features is an NP‐hard problem which is a significantly more complex problem than distance‐based graph search approaches. This is due to nonlinear relations between the features compared with the notion of physical distance which can be measured by accumulating the subdistances in the search space.

### 4.9 Time and memory complexity analysis

A brute force implementation of the proposed search algorithm would require multiple calculations for every attribute to be performed repeatedly due to the overlap among information clusters. The time complexity of mutual interaction information is exponential in the number of variables (*O*(*n* ^2^), *n* being the number of voxels in the search space). Therefore, repeating its calculations can make the search significantly slow. However, by exploiting the existing overlaps among information clusters, we proposed an efficient implementation where, if needed, each of these calculations only takes place once for each voxel. Furthermore, by considering the spatial proximity of voxels, that is, calculating the interaction between voxels within clusters rather than the entire search space, the analysis time is further reduced. As a result, the time complexity is asymptotically smaller than *O*(*n* ^2^), which is the worst‐case scenario. The majority of calculation of the spectral relevance analysis is calculated prior to search by avoiding repetition of the matrix multiplications in Equation (9). Time complexity of spectral intragroup selection with *n* dimensions (in our case, voxels) is *O*(*n*). Another point worth mentioning is that the redundancy analysis is only performed after the set of high quality neighbors at each proximity layer are admitted to the feature cluster, which facilitates a more rapid traversal in the search space. The worst‐case scenario happens when the algorithm visits every voxel for discovering each information cluster, meaning that the entire search space increases the information of the initial cluster. However, in practice, this is a rare case.

**Figure 10:**
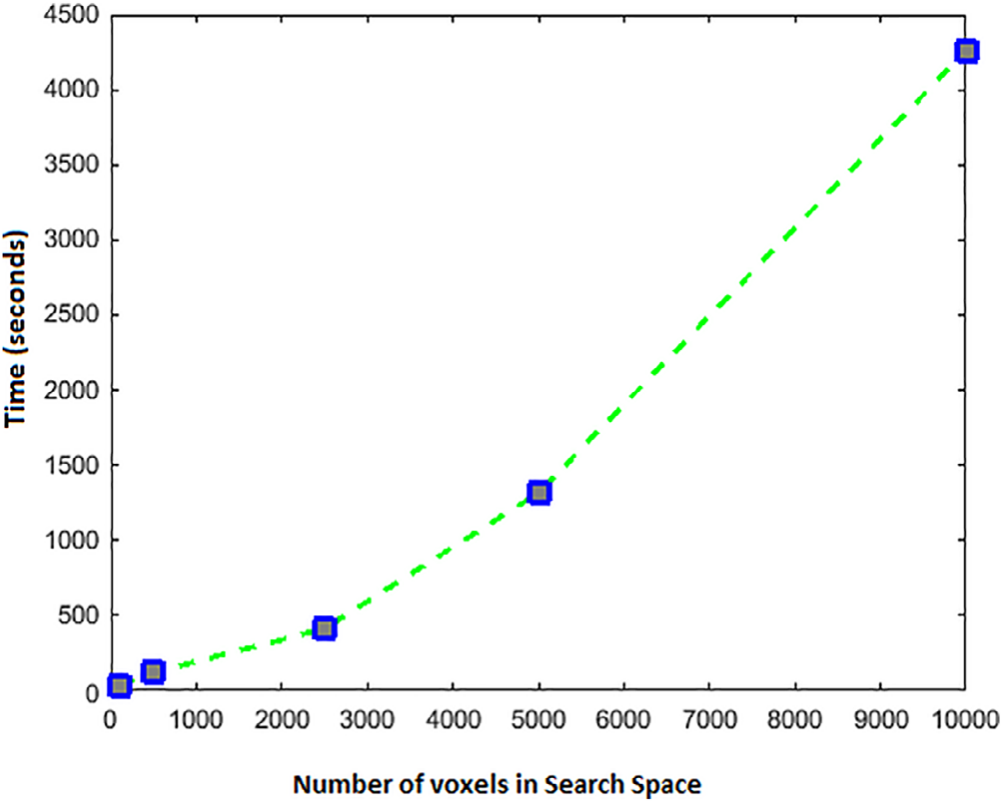
Computation time of ICS on five data set sizes

In fact, our empirical results showed that this algorithm traverses a much smaller subspace of the search space, which increases the sparsity of the interaction matrices, therefore reducing its time complexity. Nevertheless, since the algorithm is completely data driven, its computation time on a given dataset highly depends on the data itself. However, the algorithms run time in our heaviest experimental setup, which was the whole‐brain analysis for the entire dataset, was conducted within few hours, which is within the norm of feasible analysis, and significantly faster than the training or inference time of a multidimensional CNN. The computation time of ICS based on our experimental setup on datasets of five different sizes are illustrated in Figure 10.

As discussed above, ICS takes advantage of prior calculation of matrices as well as the regional calculations to decrease the number of permutations in computation. The memory requirements of the prior calculations are directly dependent on the size of the area to be searched. For the worst‐case scenario, which is when the mutual information between every other voxel is required, a nonsparse *n* × *n* matrix is created where *n* is the number of voxels in the entire search space. However, as mentioned previously, the regional information calculations in ICS normally generates a sparse matrix.

### 4.10 Limitations

Despite the mentioned advantages, ICS bears certain limitations which we point out in this section.

In ICS, the heuristic measure for cluster expansion is the discrimination power of the combined information of a group of contiguous voxels between groups of subjects. This calculation is appropriate for group level analysis, thus imposing a limitation to ICS for subject level fMRI analysis which is also desirable due to its consideration of personal variations.

Another limitation of this approach is the fact that the activation time series are averaged to facilitate the search through the single values for each voxel. This transformation ignores the temporal dynamics among the active regions. Future improvements to the proposed approach can consider the temporal dynamics of activation for detection of clusters of information.

Moreover, the case study data used in this work was derived from resting state fMRI, which constitutes only one type of MVPA. Task fMRI in which temporally separated events are modeled, is another commonly analyzed MVPA case. However, a large enough dataset of task fMRI corresponding to different neurological conditions for such analysis is difficult to obtain. As more datasets are collected or available publicly, this issue can be mitigated in the future.

Another current limitation of ICS is the fact that its present design is only applicable to cross‐sectional studies. As a future extension of this approach, this approach can be incorporated into multimodal studies where features are not limited to one imaging modality.

Reducing the discussed limitations requires further methodological explorations and logistical improvements. As future work, our objective will include formulation of temporal dynamics of functional connectivity as well as proposing subject level information measures.

## Supporting information


**Appendix** **S1:** Supporting information

## Data Availability

The dataset used in this paper is from the the Autism Brain Imaging Data Exchange (ABIDE) initiative (URL: http://fcon_1000.projects.nitrc.org/indi/abide/).

## References

[hbm24944-bib-0001] Aliferis, C. F. , Statnikov, A. , Tsamardinos, I. , Mani, S. , & Koutsoukos, X. D. (2010). Local causal and Markov blanket induction for causal discovery and feature selection for classification part I: Algorithms and empirical evaluation. Journal of Machine Learning Research, 11, 171–234.

[hbm24944-bib-0002] Balakrishnama, S. , & Ganapathiraju, A. (1998). Linear discriminant analysis‐a brief tutorial. Institute for Signal and Information Processing, 18, 1–8.

[hbm24944-bib-0003] Baron‐Cohen, S. , Ring, H. A. , Bullmore, E. T. , Wheelwright, S. , Ashwin, C. , & Williams, S. C. (2000). The amygdala theory of autism. Neuroscience & Biobehavioral Reviews, 24(3), 355–364. 10781695 10.1016/s0149-7634(00)00011-7

[hbm24944-bib-0004] Bauman, M. , & Kemper, T. L. (1985). Histoanatomic observations of the brain in early infantile autism. Neurology, 35(6), 866–866. 4000488 10.1212/wnl.35.6.866

[hbm24944-bib-0005] Belkin, M. , & Niyogi, P. (2003). Laplacian eigenmaps for dimensionality reduction and data representation. Neural Computation, 15(6), 1373–1396.

[hbm24944-bib-0006] Bjo¨rnsdotter, M. , Rylander, K. , & Wessberg, J. (2011). A Monte Carlo method for locally multivariate brain mapping. Neuroimage, 56(2), 508–516. 20674749 10.1016/j.neuroimage.2010.07.044

[hbm24944-bib-0007] Brown, G. (2009). A new perspective for information theoretic feature selection. Artificial Intelligence and Statistics, 5, 49–56.

[hbm24944-bib-0008] Cascio, C. J. , Foss‐Feig, J. H. , Heacock, J. L. , Newsom, C. R. , Cowan, R. L. , Benningfield, M. M. , … Cao, A. (2012). Response of neural reward regions to food cues in autism spectrum disorders. Journal of Neurodevelopmental Disorders, 4(1), 9. 22958533 10.1186/1866-1955-4-9 PMC3436657

[hbm24944-bib-0009] Chen, Y. , Namburi, P. , Elliott, L. T. , Heinzle, J. , Soon, C. S. , Chee, M. W. , & Haynes, J. D. (2011). Cortical surface‐based searchlight decoding. Neuroimage, 56(2), 582–592. 20656043 10.1016/j.neuroimage.2010.07.035

[hbm24944-bib-0010] Davis, T. , LaRocque, K. F. , Mumford, J. , Norman, K. A. , Wagner, A. D. , & Poldrack, R. A. (2014). What do differences between multi‐voxel and univariate analysis mean? How subject‐, voxel‐, and trial‐level variance impact fMRI analysis. Neuroimage, 97, 271–283. 24768930 10.1016/j.neuroimage.2014.04.037 PMC4115449

[hbm24944-bib-0011] Dechter, R. , & Pearl, J. (1985). Generalized best‐first search strategies and the optimality of A. Journal of the ACM (JACM), 32(3), 505–536.

[hbm24944-bib-0012] DeVore, R. A. , & Temlyakov, V. N. (1996). Some remarks on greedy algorithms. Advances in Computational Mathematics, 5(1), 173–187.

[hbm24944-bib-0013] Di Martino, A. , Yan, C.‐G. , Li, Q. , Denio, E. , Castellanos, F. X. , Alaerts, K. , … Milham, M. P. (2014). The autism brain imaging data exchange: Towards a large‐scale evaluation of the intrinsic brain architecture in autism. Molecular Psychiatry, 19(6), 659. 23774715 10.1038/mp.2013.78 PMC4162310

[hbm24944-bib-0014] Etzel, J. A. , Zacks, J. M. , & Braver, T. S. (2013). Searchlight analysis: Promise, pitfalls, and potential. Neuroimage, 78, 261–269. 23558106 10.1016/j.neuroimage.2013.03.041 PMC3988828

[hbm24944-bib-0015] Fatemi, S. H. , Aldinger, K. A. , Ashwood, P. , Bauman, M. L. , Blaha, C. D. , Blatt, G. J. , … Welsh, J. P. (2012). Consensus paper: Pathological role of the cerebellum in autism. The Cerebellum, 11(3), 777–807. 22370873 10.1007/s12311-012-0355-9 PMC3677555

[hbm24944-bib-0016] Gardumi, A. , Ivanov, D. , Hausfeld, L. , Valente, G. , Formisano, E. , & Uludağ, K. (2016). The effect of spatial resolution on decoding accuracy in fMRI multivariate pattern analysis. Neuroimage, 132, 32–42. 26899782 10.1016/j.neuroimage.2016.02.033

[hbm24944-bib-0017] Gramfort, A. , Thirion, B. , & Varoquaux, G. (2013). *Identifying predictive regions from fMRI with TV‐L1 prior*. In Pattern Recognition in Neuroimaging (PRNI), 2013 International Workshop on IEEE. (pp. 17–20).

[hbm24944-bib-0018] G'Sell, M. G. , Wager, S. , Chouldechova, A. , & Tibshirani, R. (2016). Sequential selection procedures and false discovery rate control. Journal of the Royal Statistical Society: Series B (Statistical Methodology), 78(2), 423–444.

[hbm24944-bib-0019] Heinsfeld, A. S.´l. , Franco, A. R. , Craddock, R. C. , Buchweitz, A. , & Meneguzzi, F. (2018). Identification of autism spectrum disorder using deep learning and the ABIDE dataset. NeuroImage: Clinical, 17, 16–23. 29034163 10.1016/j.nicl.2017.08.017 PMC5635344

[hbm24944-bib-0020] Heller, R. , Stanley, D. , Yekutieli, D. , Rubin, N. , & Benjamini, Y. (2006). Cluster‐based analysis of FMRI data. NeuroImage, 33(2), 599–608. 16952467 10.1016/j.neuroimage.2006.04.233

[hbm24944-bib-0021] Hossain, M. S. , Umar, S. , Alsulaiman, M. , & Muhammad, G. (2019). Applying deep learning for epilepsy seizure detection and brain mapping visualization. ACM Transactions on Multimedia Computing, Communications, and Applications (TOMM), 15(1s), 10.

[hbm24944-bib-0022] Huettel, S. A. , Song, A. W. , McCarthy, G. (2004). Functional magnetic resonance imaging (Vol. 1). Sunderland, MA: Sinauer Associates.

[hbm24944-bib-0023] Jang, H. , Plis, S. M. , Calhoun, V. D. , & Lee, J.‐H. (2017). Task‐specific feature extraction and classification of fMRI volumes using a deep neural network initialized with a deep belief network: Evaluation using sensorimotor tasks. NeuroImage, 145, 314–328. 27079534 10.1016/j.neuroimage.2016.04.003 PMC5064875

[hbm24944-bib-0024] Jimura, K. , & Poldrack, R. A. (2012). Analyses of regional‐average activation and multivoxel pattern information tell complementary stories. Neuropsychologia, 50(4), 544–552. 22100534 10.1016/j.neuropsychologia.2011.11.007

[hbm24944-bib-0025] Kamnitsas, K. , Ledig, C. , Newcombe, V. F. J. , Simpson, J. P. , Kane, A. D. , Menon, D. K. , … Glocker, B. (2017). Efficient multi‐scale 3D CNN with fully connected CRF for accurate brain lesion segmentation. Medical Image Analysis, 36, 61–78. 27865153 10.1016/j.media.2016.10.004

[hbm24944-bib-0026] Koller, D. , & Sahami, M. (1996). Toward optimal feature selection. Technical Report. Stanford InfoLab.

[hbm24944-bib-0027] Kriegeskorte, N. , & Bandettini, P. (2007). Analyzing for information, not activation, to exploit high‐resolution fMRI. NeuroImage, 38(4), 649–662. 17804260 10.1016/j.neuroimage.2007.02.022 PMC2099257

[hbm24944-bib-0028] Kriegeskorte, N. , Goebel, R. , & Bandettini, P. (2006). Informationbased functional brain mapping. Proceedings of the National Academy of Sciences of the United States of America, 103(10), 3863–3868. 16537458 10.1073/pnas.0600244103 PMC1383651

[hbm24944-bib-0029] Liu, J. , Pan, Y. , Li, M. , Chen, Z. , Tang, L. , Lu, C. , & Wang, J. (2018). Applications of deep learning to MRI images: A survey. Big Data Mining and Analytics, 1(1), 1–18.

[hbm24944-bib-0030] Lu, Y. , Jiang, T. , & Zang, Y. (2003). Region growing method for the analysis of functional MRI data. NeuroImage, 20(1), 455–465. 14527606 10.1016/s1053-8119(03)00352-5

[hbm24944-bib-0031] Mohar, B. , Alavi, Y. , Chartrand, G. , Oellermann, O. R. , & Schwenk, A. J. (1991). The Laplacian spectrum of graphs In Graph theory, combinatorics, and applications (Vol. 2 p. 871–898). Wiley.

[hbm24944-bib-0032] Ng, B. , & Abugharbieh R. (2011). *Generalized sparse regularization with application to fMRI brain decoding*. In Biennial International Conference on Information Processing in Medical Imaging. Springer. (pp. 612–623). 10.1007/978-3-642-22092-0_50 21761690

[hbm24944-bib-0033] Nie, F. , Xiang, S. , Jia, Y. , Zhang, C. , & Yan, S. (2008). Trace ratio criterion for feature selection. AAAI, 2, 671–676.

[hbm24944-bib-0034] Norman, K. A. , Polyn, S. M. , Detre, G. J. , & Haxby, J. V. (2006). Beyond mind‐reading: Multi‐voxel pattern analysis of fMRI data. Trends in Cognitive Sciences, 10(9), 424–430. 16899397 10.1016/j.tics.2006.07.005

[hbm24944-bib-0035] Perkins, S. , & Theiler, J. (2003). *Online feature selection using grafting*. In Proceedings of the 20th International Conference on Machine Learning (ICML‐03). (pp. 592–599).

[hbm24944-bib-0036] Pinaya, W. H. L. , Gadelha, A. , Doyle, O. M. , Noto, C. , Zugman, A. , Cordeiro, Q. , … Sato, J. R. (2016). Using deep belief network modelling to characterize differences in brain morphometry in schizophrenia. Scientific Reports, 6, 38897. 27941946 10.1038/srep38897 PMC5151017

[hbm24944-bib-0037] Preparata, F. P. , & Shamos, M. I. (2012). Computational geometry: An introduction. New York, NY: Springer Science & Business Media.

[hbm24944-bib-0038] Ramos, T. C. , Balardin, J. B. , Sato, J. R. , & Fujita, A. (2018). Abnormal cortico‐cerebellar functional connectivity in autism spectrum disorder. Frontiers in Systems Neuroscience, 12, 74. 30697151 10.3389/fnsys.2018.00074 PMC6341229

[hbm24944-bib-0039] Rosenblatt, J. D. , Finos, L. , Weeda, W. D. , Solari, A. , & Goeman, J. J. (2018). All‐resolutions inference for brain imaging. Neuroimage, 181, 786–796. 30056198 10.1016/j.neuroimage.2018.07.060

[hbm24944-bib-0040] Rousseeuw, P. J. (1987). Silhouettes: A graphical aid to the interpretation and validation of cluster analysis. Journal of Computational and Applied Mathematics, 20, 53–65.

[hbm24944-bib-0041] Sarraf, S. , & Tofighi, G. (2016). Classification of Alzheimer's disease using fMRI data and deep learning convolutional neural networks. arXiv Preprint arXiv, 1603, 08631.

[hbm24944-bib-0042] Schumann, C. M. , Hamstra, J. , Goodlin‐Jones, B. L. , Lotspeich, L. J. , Kwon, H. , Buonocore, M. H. , … Amaral, D. G. (2004). The amygdala is enlarged in children but not adolescents with autism; the hippocampus is enlarged at all ages. Journal of Neuroscience, 24(28), 6392–6401. 15254095 10.1523/JNEUROSCI.1297-04.2004 PMC6729537

[hbm24944-bib-0043] Shimizu, Y. , Yoshimoto, J. , Toki, S. , Takamura, M. , Yoshimura, S. , Okamoto, Y. , … Doya, K. (2015). Toward probabilistic diagnosis and understanding of depression based on functional MRI data analysis with logistic group LASSO. PLoS One, 10(5), e0123524. 25932629 10.1371/journal.pone.0123524 PMC4416710

[hbm24944-bib-0044] Stelzer, J. , Chen, Y. , & Turner, R. (2013). Statistical inference and multiple testing correction in classification‐based multi‐voxel pattern analysis (MVPA): Random permutations and cluster size control. NeuroImage, 65, 69–82. 23041526 10.1016/j.neuroimage.2012.09.063

[hbm24944-bib-0045] Swearingen, J. (2015). Characterizing the temporal dynamics in functional connectivity measured with fMRI. South Carolina: ProQuest Dissertations Publishing.

[hbm24944-bib-0046] Tan, G.‐Z. , He, H. , & Aaron, S. (2006). Global optimal path planning for mobile robot based on improved Dijkstra algorithm and ant system algorithm. Journal of Central South University of Technology, 13(1), 80–86.

[hbm24944-bib-0047] Tee, J. , & Taylor, D. P. (2018). Is information in the brain represented in continuous or discrete form? arXiv:1805.01631 [q‐bio.NC].

[hbm24944-bib-0048] Tibshirani, R. , & Saunders, M. (2005). Sparsity and smoothness via the fused lasso. Journal of the Royal Statistical Society: Series B (Statistical Methodology), 67(1), 91–108.

[hbm24944-bib-0049] Toiviainen, P. , Alluri, V. , Brattico, E. , Wallentin, M. , & Vuust, P. (2014). Capturing the musical brain with lasso: Dynamic decoding of musical features from fMRI data. Neuroimage, 88, 170–180. 24269803 10.1016/j.neuroimage.2013.11.017

[hbm24944-bib-0050] Traut, N. , Beggiato, A. , Bourgeron, T. , Delorme, R. , Rondi‐Reig, L. , Paradis, A. L. , & Toro, R. (2018). Cerebellar volume in autism: Literature metaanalysis and analysis of the autism brain imaging data exchange cohort. Biological Psychiatry, 83(7), 579–588. 29146048 10.1016/j.biopsych.2017.09.029

[hbm24944-bib-0051] Uddin, L. Q. , Menona, V. , Young, C. B. , Ryali, S. , Chen, T. , Khouzam, A. , … Hardan, A. Y. (2011). Multivariate searchlight classification of structural magnetic resonance imaging in children and adolescents with autism. Biological Psychiatry, 70(9), 833–841. 21890111 10.1016/j.biopsych.2011.07.014 PMC3191298

[hbm24944-bib-0052] Vafaie, H. , & Imam, I. F. (1994). *Feature selection methods: genetic algorithms vs. greedy‐like search*. In Proceedings of the International Conference on Fuzzy and Intelligent Control Systems. Vol. 51, p. 28.

[hbm24944-bib-0053] Varol, E. , Sotiras, A. , & Davatzikos, C. (2018). MIDAS: Regionally linear multivariate discriminative statistical mapping. NeuroImage, 174, 111–126. 29524624 10.1016/j.neuroimage.2018.02.060 PMC5949280

[hbm24944-bib-0054] Wang, J. , Wang, M. , Li, P. , Liu, L. , Zhao, Z. , Hu, X. , & Wu, X. (2015). Online feature selection with group structure analysis. IEEE Transactions on Knowledge and Data Engineering, 27(11), 3029–3041.

[hbm24944-bib-0055] Wang, X. , Liang, X. , Jiang, Z. , Nguchu, B. A. , Zhou, Y. , Wang, Y. , … Qiu, B. (2019). Decoding and mapping task states of the human brain via deep learning. Human Brain Mapping, XX, XX. 10.1002/hbm.24891 PMC7267978 31816152

[hbm24944-bib-0056] Welling, M. (2005). *Fisher linear discriminant analysis*. In Department of Computer Science, University of Toronto 3.1.

[hbm24944-bib-0057] Wilks, D. S. (2011). Cluster analysis In International geophysics (Vol. 100, pp. 603–616). Cambridge, MA: Elsevier.

[hbm24944-bib-0058] Wolz, R. , Aljabar, P. , Hajnal, J. V. , & Rueckert, D. (2010). *Manifold learning for biomarker discovery in MR imaging*. In International Workshop on Machine Learning in Medical Imaging. Springer. (pp. 116–123).

[hbm24944-bib-0059] Wong, A. C.‐N. , Palmeri, T. J. , Rogers, B. P. , Gore, J. C. , & Gauthier, I. (2009). Beyond shape: How you learn about objects affects how they are represented in visual cortex. PLoS One, 4(12), e8405. 20027229 10.1371/journal.pone.0008405 PMC2794531

[hbm24944-bib-0060] Wu, X. , Yu, K. , Hefei, W. D. , Wang, H. , & Zhu, X. (2013). Online feature selection with streaming features. IEEE Transactions on Pattern Analysis and Machine Intelligence, 35(5), 1178–1192. 23520258 10.1109/TPAMI.2012.197

[hbm24944-bib-0061] Zhao, Z. , & Liu, H. (2007). *Spectral feature selection for supervised and unsupervised learning*. In Proceedings of the 24th International Conference on Machine Learning. ACM, pp. 1151–1157.

[hbm24944-bib-0062] Zhou, J. , Foster, D. , Stine, R. , Ungar, L. (2005). *Streaming feature selection using alpha‐investing*. In Proceedings of the 11th ACM SIGKDD International Conference on Knowledge Discovery in Data Mining. ACM (pp. 384–393).

[hbm24944-bib-0063] Zhou, Y. , Shi, L. , Cui, X. , Wang, S. , & Luo, X. (2016). Functional connectivity of the caudal anterior cingulate cortex is decreased in autism. PLoS One, 11(3), e0151879. 26985666 10.1371/journal.pone.0151879 PMC4795711

