# Supplementary material for: A heuristic information cluster search approach for precise functional brain mapping
Source: Hum Brain Mapp. 2020 Feb 7;41(9):2263–80. doi: 10.1002/hbm.24944 (PMC7267912; doi:10.1002/hbm.24944)
Supplement: Supplementary file 1 — Appendix S1: Supporting information [file HBM-41-2263-s001.docx]

**Supplementary Material**

# 0.1. Data Preparation

Since ICS performs a search among neighbors of the clusters, it is necessary to create the neighborhood matrix of the voxels before starting the search procedure, and just point the algorithm to the neighbors of the voxel during the search process. The neighborhood matrix can be created by simply measuring the euclidean distance of the voxels based on their three dimensional coordinates. In this matrix, called matrix *M_neighbors_*, each row belongs to a voxel, and the coluics include the indices of their immediate neighboring voxels. Note that topological properties of specific regions can be considered during pre-processing using FMRIB Software Library (FSL), and then the neighborhood matrix can be created. This would not affect the performance of the search, as it only requires the indices of the neighboring voxels.

# 0.2. ICS pseudocode

Here we explain the steps of ICS in more detail through the pseudocode provided in Algorithm 1. As can be seen in the pseudo code, the input to the proposed approach includes an *M* × *P* matrix *X* where *M* is the number of subjects and *P* is the number of search space voxels, a vector

*Y* containing the labels, and the *M_neighbors_* neighborhood matrix explained in the above section. Each element *X_ij_* contains the averaged time course of voxel *j* for subject *i* over time. Note that the input to this algorithm can include more than two groups, and similar setup can be designed for multi-class scenario. As the search starts, the spectral discriminant score of

Cluster *C_temp_*, which initially only includes the voxel *V_start_* is measured by exploiting the pre-calculated local and global affinity matrix multiplications based on equation 5 in the main text (line 8 of the pseudocode). Then, for each neighboring voxel of *C_temp_*, the spectral discriminant score of the addition of the neighbors *V_neighbor_* and *C_temp_* are calculate and compared with the score of *C_temp_*, and useful voxels are added to *C_temp_* to expand it (lines 9 to 20). After one step of neighborhood search and expansion of *C_temp_*, redundancy criteria is performed by first looking up the matrices that will contain the calculations based on the equations 8 and 9 for each voxel. If the corresponding values for the voxels inside *C_temp_* do exist in matrix *Interact_u_*, (meaning that the voxel has been previously visited), the values are directly used to avoid recalculations. Otherwise, the mutual information elements are

1

calculated and placed in its corresponding location in that matrix for future use (lines 23 to 27).

The redundancy scores of each voxel inside *C_temp_* is then compared with a pre-specified threshold *T* to remove redundant features (lines 28 to 30). Note that *T* can be assigned in various fashions depending on how strict the redundancy analysis is preferred to be. Also, in this algorithm we avoid removing the recently added voxels during the redundancy step to avoid the possibility of falling into infinite loops by repeatedly adding and removing the same voxels (note the loop condition in line 21 which excludes the newly added voxels). For the same reason, we avoid adding newly removed voxels (note the condition in line 10). If the algorithm does not find any more useful neighbors during the search, after saving the results in the output it moves to the voxel next to *V_start_*, and pursues the same steps until it covers the entire search space, providing a complete list of information clusters and their information value (lines 34 and 35).

2

**Algorithm 1** Neighborhood search with DP

1: **Input:** Data *X*_(_*_m_*_×_*_p_*_)_ (*m* :number of subjects and *p* : number of voxels in search space); Labels vector *Y*_(_*_m_*_×1)_; Matrix *M_neighbors_* with *m* rows

2: **Output:** Matrix *C_out_* with *m* rows; vector *I_out_*_(_*_m_*_×1)_

3: Calculate *M_w_* and *M_s_ .* Matrix multiplying for local and global affinity

4: (*Interact_u_,N_array_,F_array_*) ←∅; *. Interact_u_*:interaction matrix, *N_array_*: helpful neighbors, *F_array_*: redundant neighbors

5: *V_start_*← 1; *.* Starting voxel

6: *C_temp_*←X(:,V*_start_*); *.* Starting cluster 7: **while** *V_start_* ≤ *p* **do** *.* Start the search

8: Calculate *S*(*C_temp_*) (spectral discriminant score) via *M_w_* and *M_s_*;

9: **for** ∀v*neighbor*∈M*neighbors*(*Ctemp*) **do** *.* Relevance analysis 10: **if** *v_neighbor_* ∈6 *F_array_* **then** *.* Avoid redundant voxels of last step

11: Calculate *S*(*Ctemp* S*vneighbor*) via *Mw* and *Ms*;

12: **if** *S*(*Ctemp* S*vneighbor*) *> S*(*Ctemp*) **then**

13: *Narray* ← *Narray* S*vneighbor*;

14: *Counter* + +;

15: **end if**

16: **end if**

17: **end for**

18: *Farray* ←∅; *.* Empty *Farray*

19: **if** *Counter >* 0 **then** *.* There are helpful neighbors

20: *Ctemp* ← *Ctemp* S*X*(:*,Narray*);

21: **for** ∀v∈C*_temp_* − *N_array_* **do** *.* Redundancy analysis;

22: **if** *Feature_inf_*(*v*) 6= 0 **then**

23: Look up *Interact_u_* to calculate redundancy *J* for *v*;

24: **end if**

25: **if** *Feature_inf_*(*v*) = 0 **then**

26: Calculate score *J* for voxel *v* and store them in *Interact_u_*.;

| 27: | **end if** |  |
| --- | --- | --- |
| 28: | **if** *J < T* **then** | *. T* is a pre-specified threshold |
| 29: | *Farray* ← *Farray* S*v*; |  |
| 30: | **end if** |  |
| 31: | **end for** |  |
| 32: | *Ctemp* ← *Ctemp* − *Farray*; | *.* Remove redundancies |
| 33: | **if** *Counter* = 0 **then** | *.* No helpful neighbors found |
| 34: | *Cout*(*Vstart*);← *Ctemp*; | *.* Output results |
| 35: | *Iout*(*Vstart*) ←S; | *.* Output results |
| 36: | *Vstart* = *Vstart* + 1; |  |
| 37: | *Ctemp*←X(:,V*start*); | *.* Start a new cluster |

38: **end if**

39: *Counter* ← 0;

40: *N_array_* ←∅;

41: *Cluster* ← *Cluster* ^S^*X*(:*,N_array_*);

42:

43: **return** [*C_out_,I_out_*];

# 0.3. Additional prediction results


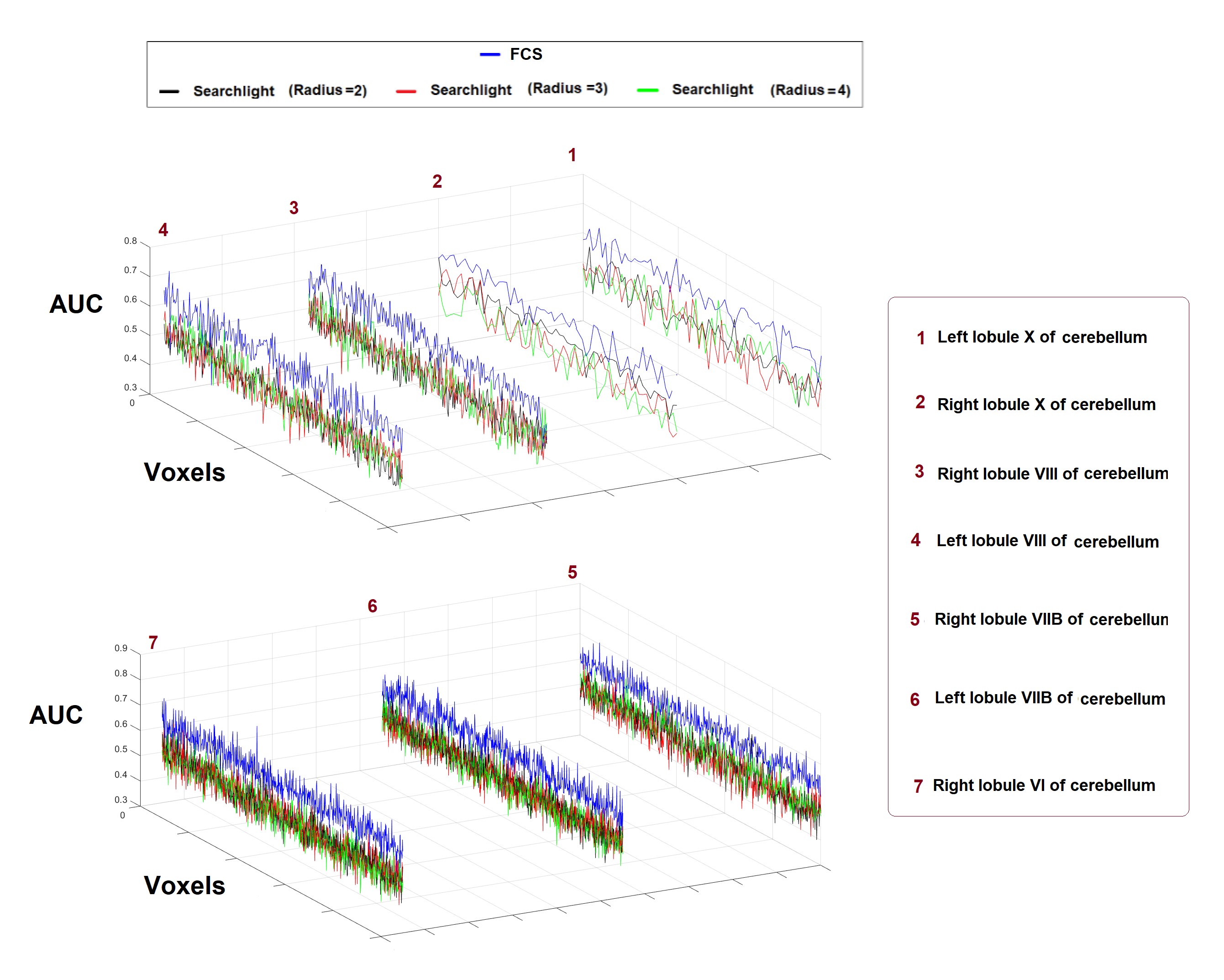


Figure 1: Prediction results of regions of interest based on AAL atlas. The experimental setup and procedure are similar to the result section in the main text.

4
